# Whole-body synchronization in autistic adults

**DOI:** 10.3389/fnhum.2026.1866968

**Published:** 2026-07-31

**Authors:** Tim Schnitzler, Raphael Perla, Christoph W. Korn

**Affiliations:** Department of Psychiatry and Psychotherapy, Department of Psychosocial Medicine, Psychotherapy and Family Health, Heidelberg University, Heidelberg, Germany

**Keywords:** affect, body movement, interpersonal synchronization, motor synchronization, self-regulation

## Abstract

**Introduction:**

A characteristic feature of autistic individuals is deviations in nonverbal behavior during social interactions, evident in altered synchronization. In non-autistic individuals, increased synchronization adversely affects the capacity for emotional self-regulation. Yet, synchronization in autistic individuals has primarily been examined in conversational contexts.

**Methods:**

In this study, 33 autistic and 33 non-autistic adults performed a dyadic movement task. Participants were asked to “have a conversation without words” using only improvised movements. Using an inertial sensor-based motion capture system, we investigated interpersonal synchronization.

**Results:**

Our findings indicated greater synchronization in autistic dyads than in non-autistic dyads. Mixed dyads did not differ from either autistic or non-autistic dyad. Across all three dyad types, the task was not associated with reliable observed changes in emotional self-regulation, positive affect, or negative affect, and baseline-adjusted post-task outcomes were not reliably associated with interpersonal synchronization. However, it is important to note our small sample size, which limits the robustness and generalizability of the results.

**Discussion:**

We discuss our findings in relation to the task demands. Whole-body synchronization played a crucial role in our study, unlike in previous studies of conversational settings. Consequently, our task relied less on language, familiarity with social interaction tasks, and eye contact, all of which are aspects that autistic individuals often find challenging. Our findings contribute to the ongoing debate on interpersonal synchronization in autism spectrum condition by extending previous studies on conversational contexts to whole-body movement. The data can be combined with future datasets to create larger samples and provide a more nuanced understanding about synchronization in autism spectrum condition.

## 1 Introduction

Interpersonal synchronization arises in social interactions. It encompasses phenomena defined by the spontaneous rhythmic and transient coordination of behaviors, emotions, or other processes among participants (Bernieri and Rosenthal, 1991). It includes both conscious and unconscious coordination processes that enable people to synchronize their actions, gestures and rhythms. Interpersonal synchronization has been attributed a key role in the development of various social behaviors in non-autistic individuals, such as prosocial behavior, positive affect and social cognition (Mogan et al., 2017; Ayache et al., 2021). In particular, the positive correlation between interpersonal synchronization and positive affect in non-autistic individuals has been demonstrated in numerous meta-analyses. The degree of interpersonal synchronization among individuals predicts positive interaction outcomes such as enjoyment of interaction and a sense of connection (Mogan et al., 2017; Vicaria and Dickens, 2016). Interpersonal synchronization can manifest at multiple levels, including the neural level (Turk et al., 2022), the physiological level (Pérez et al., 2021), or during a dyadic movement improvisation task when two people engage in a “conversation without words” (Galbusera et al., 2019). Another key aspect is the role of motor synchronization in developing and preserving social skills. Even in early childhood, synchronized interaction patterns emerge between caregivers and children, serving as the foundation for attachment and communicative development (Feldman, 2007). Interruptions in these processes are associated to a variety of clinical conditions, such as autism spectrum condition, highlighting the clinical significance of this area of research (Fitzpatrick et al., 2016).

Because challenges in social communication are a core feature of autism spectrum condition (American Psychiatric Association, 2013), the analysis of interpersonal synchronization is a promising approach to investigating autism. For a long time, research on autism spectrum condition focused primarily on social cognition (Polónyiová et al., 2024; Baron-Cohen, 1995). While such an approach takes intersubjectivity into account, it still views autism spectrum condition from an individualistic perspective, attributing the intersubjective difficulties to the individual. In recent years, researchers have increasingly studied interactions between autistic and non-autistic individuals (Schilbach et al., 2013). This shift has broadened the focus to interaction, because successful interaction depends on all participants (Milton, 2012; Schnitzler and Fuchs, 2025). As a result, it has been acknowledged that social challenges are encountered by both autistic and non-autistic individuals (Heasman and Gillespie, 2018).

Variations in interpersonal synchronization may stem from disparities in the embodiment of autistic and non-autistic individuals, namely in intrapersonal coordination and perceptual-motor skills (De Jaegher, 2013). Due to the relevance of interpersonal synchronization processes for social interaction, these disparities have been linked to interindividual differences in social interaction skills (Fitzpatrick et al., 2017) and have been found to correlate negatively with the severity of autism symptoms (Zampella et al., 2020; Kawasaki et al., 2017). Prior research on interpersonal synchronization between autistic and non-autistic individuals has tended to reveal less interpersonal synchronization in mixed (one non-autistic and one autistic person) than in non-autistic dyads (for overviews, see Mcnaughton and Redcay, 2020; Glass and Yuill, 2024b; Carnevali et al., 2024). However, even within mixed dyads, a certain degree of interpersonal synchronization can be observed (Romero et al., 2018; Fitzpatrick et al., 2017). Only a few studies have identified equivalent or increased interpersonal synchronization among autistic children interacting with one another relative to non-autistic children (Glass and Yuill, 2024a). Depending on the task, other work has found partially comparable interpersonal synchronization when an autistic or a non-autistic adult engaged with a confederate (a university student or member of the research group) who was unaware of the participant's diagnostic group (Bierlich et al., 2025). In recent years, researchers have studied interpersonal synchronization in autistic individuals in more naturalistic situations. In conversational situations, autistic children (Zampella et al., 2020) and autistic adults showed lower interpersonal synchronization than the non-autistic group or a group of patients who received another psychiatric diagnosis during diagnostic interviews rather than a diagnosis of autism spectrum condition (Georgescu et al., 2020; Koehler et al., 2022). Evidence suggests that the paradigm influences interpersonal synchronization. Autistic individuals more readily synchronize spontaneously with others than when attempting to do so consciously (Glass and Yuill, 2024b).

Previous research has mostly compared interpersonal synchronization in mixed and non-autistic dyads, while autistic dyads have been insufficiently investigated. This has led researchers to ascribe the origin of interpersonal synchronization issues to autistic individuals, neglecting the reality that social relationships rely on reciprocity and mutuality (Glass and Yuill, 2024a). People do not consistently interact in the same way in all contexts. Instead, individuals modify their communication patterns and approaches in accordance with variables such as cultural origins, relationships, social norms, and personal preferences (Interactional Heterogeneity Hypothesis; Georgescu et al., 2020). Since people often interact more effectively with members of the same group (Milton, 2012), it might be expected that autistic people will synchronize more easily when interacting with other autistic people than with non-autistic people. To date, only three studies have investigated this hypothesis, with conflicting results. One study showed a more pronounced interpersonal synchronization in non-autistic dyads compared to autistic and mixed dyads in several conversational tasks (Georgescu et al., 2020). Another study found no group differences among the three dyad groups (Efthimiou et al., 2025). Among adolescents, non-autistic dyad showed greater smiling synchronization than mixed dyads, while they did not differ from autistic dyad (Mcnaughton et al., 2024).

To date, studies examining interpersonal synchronization in autistic individuals in naturalistic settings have only used seated conversational contexts (Georgescu et al., 2020; Koehler et al., 2022), in which whole-body movement is less prominent than in dynamic contexts that allow individuals to move freely. Although these approaches provide valuable insights into social communication challenges, they are insufficient, as social interaction is an embodied process that involves the entire body (Eigsti, 2013). Conversations represents only one part of social interaction, whereas whole-body synchronization is particularly relevant in natural contexts such as shared movement, play, or cooperation (Wiltermuth and Heath, 2009; Biermann et al., 2023). Accordingly, the study of whole-body synchronization in dynamic settings is useful for developing a more comprehensive understanding of social interaction and interpersonal synchronization in autism spectrum condition. Furthermore, such a method provides a basis for further investigation of movement-based interventions, such as dance (Manders et al., 2022) or music therapy, which explicitly focus on promoting synchronous interaction in autism spectrum condition (Daniel et al., 2022).

It is therefore essential to study interpersonal synchronization in autistic individuals not only in conversational settings but also in dynamic situations that allow for unrestricted movement. In this context, it is particularly interesting to examine how interpersonal synchronization manifests in autistic dyads under these conditions. Autistic individuals exhibit greater connectedness and ease of interaction when communicating with other autistic individuals than with non-autistic counterparts (Crompton et al., 2020b,a). For this reason, we departed from previous studies (Koehler et al., 2022; Georgescu et al., 2020), which predominantly focus on conversational contexts. The present study used an inertial sensor-based motion tracking system. To our knowledge, this system has not previously been used with autistic individuals. It allows for free movement within a designated space.

The study also examines the relationship between interpersonal synchronization and emotional self-regulation, defined as the ability to consciously or unconsciously modify one's emotional states. Historically, interpersonal synchronization was considered to have exclusively positive effects. However, this perspective did not take into account the impact of interpersonal synchronization on the self. This question is based on hypotheses from enactive cognitive science, according to which the stability of the self requires a balance between attunement to others and the pursuit of independence from them (Kyselo and Tschacher, 2014; Varela et al., 2017). Accordingly, the human self is not understood in individualistic terms that strictly separate an agent from the environment. Rather it is conceived as an interactive dynamic. The agent actively structures its exchange with the environment, thereby generating and maintaining systemic stability. Thus, excessive interpersonal synchronization may also have negative effects on the human self, as an increased focus on the environment can limit an individual's capacity for emotional self-regulation. Galbusera et al. (2019) explored this relationship in the “Body-Conversation-Task” (BCT), in which two non-autistic individuals interacted for 5 min using only body movements. Speaking was not allowed. The synchronization assessed in this study was associated with positive affect among participants (Mogan et al., 2017; Tschacher et al., 2014), although it also resulted in increased challenges in emotional self-regulation. Thus an imbalance and a predominance of interpersonal synchronization can also have negative effects on the self. The advantage of BCT lies in its focus on whole-body movements rather than isolated body parts.

The present study aims to examine the relationship between interpersonal synchronization and emotional self-regulation in autistic individuals, a topic that has not previously been investigated. This is relevant because much of the research on interpersonal synchronization in autism spectrum condition to date has shown reduced interpersonal synchronization. However, this research has not considered the potential positive consequences of a presumed lower level of interpersonal synchronization in autism spectrum condition. One such consequences may be enhanced emotional self-regulation among autistic individuals. Emotional self-regulation among autistic individuals remains inadequately researched. Most studies have focused on children, so their findings are not strictly applicable to adults. Prior research indicates that autistic adults typically experience more challenges in emotional self-regulation than their non-autistic counterparts (Cai et al., 2018). Autistic adults use adaptive regulation strategies such as positive reappraisal less frequently and tend to use maladaptive strategies (Bruggink et al., 2016; Samson et al., 2012). However, the assessment of wheter a strategy is adaptive is based on the non-autistic population (Cai et al., 2018). In this context, it is interesting to note that avoidance strategies were negatively associated with depression in autistic adolescents (Pouw et al., 2013), although these strategies are considered maladaptive in the general population.

The present study investigates whether patterns of interpersonal synchronization differ across autistic, non-autistic, and mixed dyads during the Body Conversation Task. The study also examines task-related changes in emotional self-regulation difficulties and affect. It further examines the associations between interpersonal synchronization and baseline-adjusted post-task self-regulation difficulties and affect. Three hypotheses were proposed: *Hypothesis 1:* Non-autistic dyads will show stronger interpersonal synchronization during the Body Conversation Task compared to autistic and mixed dyads. This hypothesis is based on previous findings suggesting that non-autistic individuals display higher levels of interpersonal synchronization (Georgescu et al., 2020). We formulated this hypothesis prior data collection. Studies published since then have not found lower interpersonal synchronization in autistic dyads compared to non-autistic dyads (Efthimiou et al., 2025; Mcnaughton et al., 2024).

*Hypothesis 2.1.:* Higher levels of interpersonal synchronization will be associated with greater difficulties in self-regulation within non-autistic dyads, consistent with the findings of Galbusera et al. (2019). *Hypothesis 2.2.:* Because emotional self-regulation difficulties are considered widespread among autistic individuals, we hypothesized that increased interpersonal synchronization would lead to a smaller increase in such difficulties in autistic dyads than in non-autistic dyads. These difficulties were expected to be present independently of the interpersonal synchronization. If a ceiling effect occured, increased interpersonal synchronization might not produce a significant increase in emotional self-regulation difficulties.

*Hypothesis 3.1.:* For the non-autistic dyad, we assumed that higher levels of interpersonal synchronization would be associated with increased positive affect.

*Hypothesis 3.2.:* We did not assume such a positive correlation for the autistic dyad, as previous studies have predominantly reported lower levels of interpersonal synchronization in autistic individuals, possibly because synchronization may be experienced as less pleasant by autistic participants. Instead, for the autistic dyad, we hypothesized a positive correlation between interpersonal synchronization and negative affect. However, the relationship between interpersonal synchronization and both emotional self-regulation and affect have not been systematically investigated in autistic individuals. Hypotheses 2 and 3 are therefore based on theoretical assumptions rather than prior empirical findings.

## 2 Materials and methods

All procedures involving human participants complied with the 1964 declaration of Helsinki and its later amendments or comparable ethical standards. The ethics committee of Heidelberg University granted ethical approval (registration number: S-573/2022). The study was not preregistered.

### 2.1 Participants

The study included 33 autistic and 33 non-autistic participants (see Table 1). One mixed dyad was excluded because of missing values. The final analyzed sample therefore comprised 64 participants forming 32 dyads: 11 non-autistic dyads, 11 autistic dyads, and 10 mixed dyads. The sample size corresponded to that of another study that examined interpersonal synchronization in the three study groups (Georgescu et al., 2020). Autistic individuals were recruited at our hospital or through nearby autism centers. All autistic participants met DSM-5 diagnostic criteria for autism spectrum condition (American Psychiatric Association, 2013) and psychiatrists specializing in autism had established the diagnoses before the study. Non-autistic participants were recruited through an announcement. Inclusion criteria were age between 18 and 65 years, the ability to move the whole body easily, and knowledge of German. Exclusion criteria were pre-existing schizophrenia-spectrum disorders, bipolar disorder, substance addiction, serious internal medical conditions, or other acute psychiatric or neurological disorders. To prevent a possible influence on interpersonal synchronization, we excluded professional athletes and professional dancers. Self-reported sports activity (classified as “no sports,” “occasionally,” or “at least once a week”) did not differ significantly across participant types or dyad types.

**Table 1:** Demographic variables and diagnostic scores.

	Autistic	Non-autistic	*p*
Age	25.28 + /−7.71	25.24 + /−8.14	0.984
Sex (female/male)	9/24	9/24	1.0
MWT	29.97 + /−2.72	30.09 + /−3.93	0.885
AQ	32.94 + /−7.29	14.33 + /−6.89	< 0.001
TAS-26	50.61 + /−9.23	37.97 + /−8.33	< 0.001

Values are given as mean ± standard deviation. *p* values reflect levels of significance from independent-samples t tests. MWT: multiple-choice vocabulary test; AQ: autism quotient; TAS-26: toronto alexithymia scale−26.

Participants were assigned to one of three dyad types (see Table 1): two autistic individuals (autistic), two non-autistic individuals (non-autistic), or one autistic individual and one non-autistic individual (mixed). There were 11 dyads from each study group. Participants in each dyad were blind to their partner's diagnostic status. All three types of dyads were matched on age, sex, and verbal IQ. The matching process was based on Georgescu et al. (2020). In that study, interacting partners had the same sex (assigned at birth and based on information provided by the participants), an age difference within ± 5 years, and an IQ difference within ± 2 SD. When recruiting dyads, we made sure that the interaction partners were of the same sex and similar age. Differences in IQ were examined after testing based on the multiple-choice vocabulary test, which revealed no significant differences between the interacting partners in any of the dyads. First, we completed recruitment for autistic dyads. We then ensured that there were no differences in age or sex compared to the other two dyad types. Each participant received 27.50 euros as compensation.

### 2.2 Capturing the motion sequences

**Figure 1:**
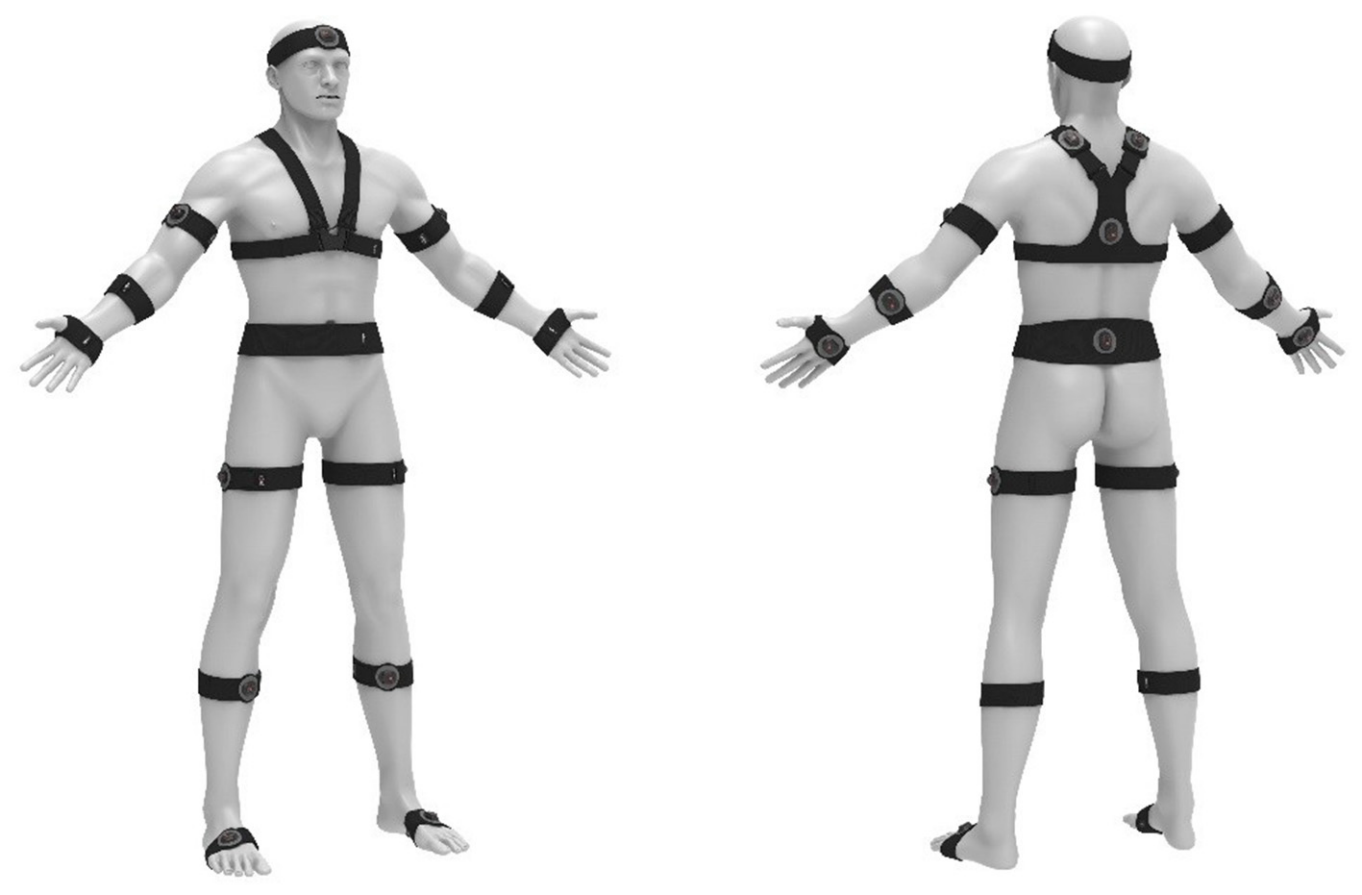
Locations of the sensors on the body. Each sensor is secured at the corresponding location with a strap. Source of the illustration: https://www.noitom.com/index.html.

We used Perception Neuron 3^®^ (PNS; Perception Neuron, Noitom, Miami, FL, USA), a lightweight, wireless, inertial sensor-based motion capture system comprising 17 sensors. Straps secured the sensors at designated body positions in accordance with PNS guidelines (see Figure 1). Throughout the measurement process, the sensors were connected to a computer via Bluetooth within an approximate 4-meter operating radius. Real-time data visualization and storage were performed using the Axis Neuron software (AXIS Neuron, Noitom, Miami, Florida, USA). The PNS was calibrated according to the manufacturer's instructions in a four-stage process. During the measurement, the translation and rotation data from each sensor were recorded and stored for each participant at a frequency of 60 Hz.

### 2.3 Setup and procedure

**Figure 2:**
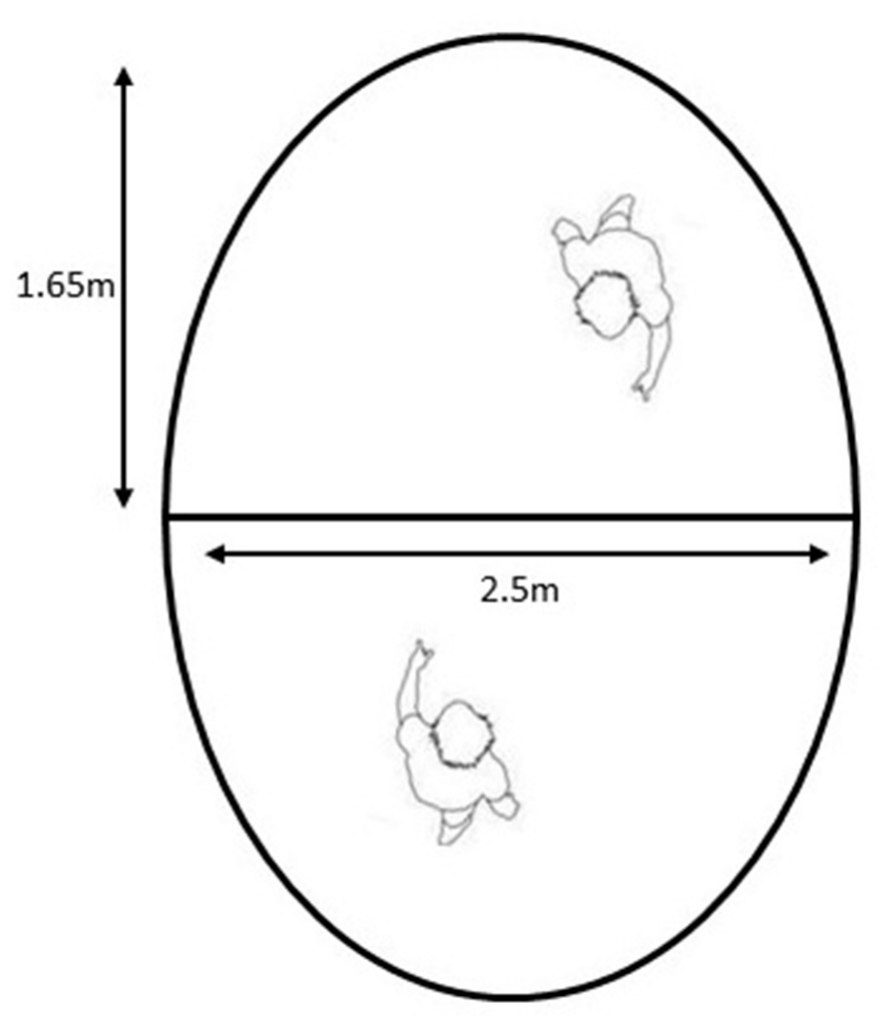
Setting of the BCT. Each participant could move freely in a semi-elliptical space measuring 2.5 m wide and 1.65 m deep. The computer was placed at one end of the center line of the ellipse.

Participants then completed questionnaires about emotional self-regulation and current affect. An ellipse measuring 2.5 m × 3.3 m was drawn on the floor and divided into two equal halves (see Figure 2). Both halves represented the area in which each participant was allowed to move during the warm-up exercise and the movement task. Lighting was kept constant throughout. After completing the questionnaires for the first time, each participant stood in one half of the ellipse, with their backs to each other. This positioning was intended to prevent unintentional interpersonal synchronization before the task. A standardized 5-min warm-up exercise for all parts of the body was performed according to Galbusera et al. (2019). The experimenter read the written instructions aloud.

The experimenter then read the instructions for the BCT, after which the PNS was calibrated in a four-step process according to the manufacturer's instructions. Once recording began, the experimenter gave the signal for the 5-min free-improvisation task. The participants were asked to “engage in a conversation without words.” They were asked to use their whole body as extensively as possible and to remain in motion. They were also asked to refrain from physical contact. Participants were permitted to move freely inside their designated area, but not to leave it. Interaction and communication were carried out exclusively via bodily movements. Throughout this period, the experimenter stayed in the room but faced away from the participants to allow for undisturbed communication. For the same reason, no video recordings were taken. After 5 min, participants completed the questionnaires on emotional self-regulation and their current affect, as well as the other questionnaires.

At the end, participants were asked about their experiences to confirm that they had followed the BCT instructions. Specifically, they were asked whether they had used whole-body movements rather than only hand movements or pantomime to communicate. All participants reported that they had completed the task in accordance with the instructions. However, these data were not systematically documented.

### 2.4 Subjective measures

Before and after the BCT, participants completed the German version of the Positive and Negative Affect Schedule to evaluate affective state (PANAS; Breyer and Bluemke, 2016) and completed the State Difficulties in Emotion Regulation Scale to assess emotional self-regulation (S-DERS; Lavender et al., 2017).

The PANAS comprises 20 items: 10 measure positive affect (e.g., excited, inspired), and 10 measure negative affect. Each item is assessed on a 5-point Likert scale. The analysis emphasized positive affect because of its significance in interpersonal synchronization. Negative affect was also included. Positive and negative affect ratings are taken as separate metrics; that is, they are typically not pooled.

The S-DERS consists of 21 items, divided into four subscales (modulate: challenges in regulating immediate emotional reactions; non-acceptance of emotions; awareness: restricted awareness of current emotions; clarity: restricted clarity of current emotions). Items are rated on a 5-point Likert scale. Higher scores indicate greater difficulties in emotion regulation. Since the S-DERS is not yet available in German, two people independently translated the English version into German and compared the wording. When the translations differed, the translators selected the wording that was clearest and closest to the original English text. A third person translated the German version back into English and compared it with the original. This comparison revealed no significant alterations. We include this German version in the Appendix. The modulate subscale assesses the pre-reflexive ability to modulate emotional reactions and the other three subscales relate to the person's reflective attitude toward their own emotions. Analogous to Galbusera et al. (2019), we therefore used the modulate subscale to assess emotional self-regulation.

Autistic traits were measured using the Autism-Spectrum Quotient (Baron-Cohen et al., 2001) and verbal IQ was assessed using a multiple-choice vocabulary test for matching purposes (Lehrl, 2005). Alexithymia was assessed using the Toronto Alexithymia Scale-−26 (Taylor et al., 1985, 1990). A basic questionnaire based on the study by Galbusera et al. (2019) was completed to document participants' physical effort in creating their “conversation without words.” Covariate descriptives and sports activity comparisons are reported in Supplemental Tables S26, S27.

### 2.5 Statistical analyses

Interpersonal synchronization was quantified from the body-movement recordings using quaternion-based movement signals across the full set of between-person segment pairings. Quaternion time series were normalized and converted to per-frame angular-speed estimates. They were then low-pass filtered with a second-order Butterworth filter (cutoff = 0.24), cleaned using an outlier rule of 20 interquartile ranges, and interpolated across missing samples. Across the 11 segments per partner (head, chest, hip, and the bilateral upper- and lower-arm and upper- and lower-leg segments), all 121 segment pairings were included. Partners' recordings were truncated to their shared analyzable frame range and segmented into non-overlapping 30-s windows (1,800 frames). Incomplete trailing frames caused by slight variations in recording duration were discarded. Within each window, cross-correlations were computed for each segment pairing across lags of up to ± 5 s. They were Fisher z-transformed, converted to absolute values, and averaged across lags and segment pairs to yield one dyad-window interpersonal synchrony score.

Observed synchrony was validated using a shuffled surrogate null. To create this null, one partner's movement signal was shuffled within 10-s blocks. The same synchrony computation was then repeated. This procedure produced a within-dyad chance benchmark.

The primary interpersonal synchronization model was estimated at the dyad-window level as

*Interpersonal synchronization*_*ij*_ = β_0_+β_1_(*dyad type*_*i*_)+*u*_*i*_+ϵ_*ij*_

where u_i_ denotes a random intercept for dyad i. This model was fitted to 321 dyad-window observations from 32 dyads (110 non-autistic windows, 110 autistic windows, and 101 mixed windows).

Questionnaire analyses were conducted at the participant level using separate models for PANAS positive affect, PANAS negative affect, S-DERS Total, and S-DERS Modulate. For task-related change and dyad-type differences in change, the primary estimand was standardized observed change, defined as the post-task minus the pre-task score. This change was modeled as a function of dyad type, mean interpersonal synchronization, and the dyad type-by-interpersonal synchronization interaction, with a random intercept for dyad. For synchronization-outcome associations, the primary estimand was the standardized post-task score adjusted for the corresponding pre-task score. The model also included dyad type, mean interpersonal synchronization across the task, the dyad type-by-interpersonal synchronization interaction, and a random intercept for dyad. These models used 64 participant-level observations per outcome (22 participants from non-autistic dyads, 22 from autistic dyads, and 20 from mixed dyads).

Both specifications were fitted and reported for each questionnaire outcome. The change-score models estimate observed pre-post change. The baseline-adjusted models estimate expected post-task scores at average pre-task score and average interpersonal synchronization. Their synchronization terms represent within-dyad-type associations between mean interpersonal synchronization and adjusted post-task scores. We report the specification most closely aligned with each question in the main text and provide the alternative specification and direct model-comparison summaries in Supplemental Table 17.

For PANAS outcomes, we also fitted a covariate robustness analysis with alexithymia entered as an additional participant-level covariate. To evaluate the model dependence of the main estimates, we compared the primary interpersonal synchronization model with models that also accounted for task interval or analyzed dyad-average synchronization (Supplemental Table 16). We also refitted singular questionnaire models without the dyad random intercept (Supplemental Table 18). In addition, we summarized model precision by calculating the minimum detectable effect for each estimand related to a hypothesis from its standard error and degrees of freedom, using a two-sided alpha of 0.05 and a target power of 80% (Supplemental Table 20). Practical-equivalence tests used two one-sided tests with equivalence bounds of ± 0.10 standardized units (Supplemental Table 19).

Full model coefficients and model fit summaries are reported in Supplemental Tables 1–4. Models were fitted in R (R Core Team, 2026) with lme4 (Bates et al., 2015), lmerTest (Kuznetsova et al., 2017), and emmeans (Lenth and Piaskowski, 2026). Supporting data handling and plotting used dplyr (Wickham et al., 2026a), readr (Wickham et al., 2026b), tidyr (Wickham et al., 2025), and ggplot2 (Wickham, 2016). Pairwise contrasts among dyad types were Tukey-adjusted within each model family. No additional across-family multiplicity correction was applied. Interpersonal synchronization group contrasts were tested using two-sided tests.

### 2.6 Community involvement

One of the first authors (TS) leads an autism consultation service focused on adult diagnosis. The service is dedicated to enhancing diagnostic practices for autistic people through further education. The results will be shared within the community in conjunction with collaborating autism centers.

## 3 Results

### 3.1 Pseudo synchronization

We first verified that the observed dyad-level synchronization exceeded a shuffled surrogate null derived from within-series 10-s shuffles. Across the 32 analyzed dyads, observed interpersonal synchronization (*M* = 0.105) was substantially higher than shuffled interpersonal synchronization (*M* = 0.054), yielding a mean difference of 0.051, *t* (31) = 32.79, *p* < 0.001, *d* = 5.80. This pattern indicates that partners' temporal coordination exceeded the shuffled chance benchmark.

### 3.2 Group differences in interpersonal synchronization

**Figure 3:**
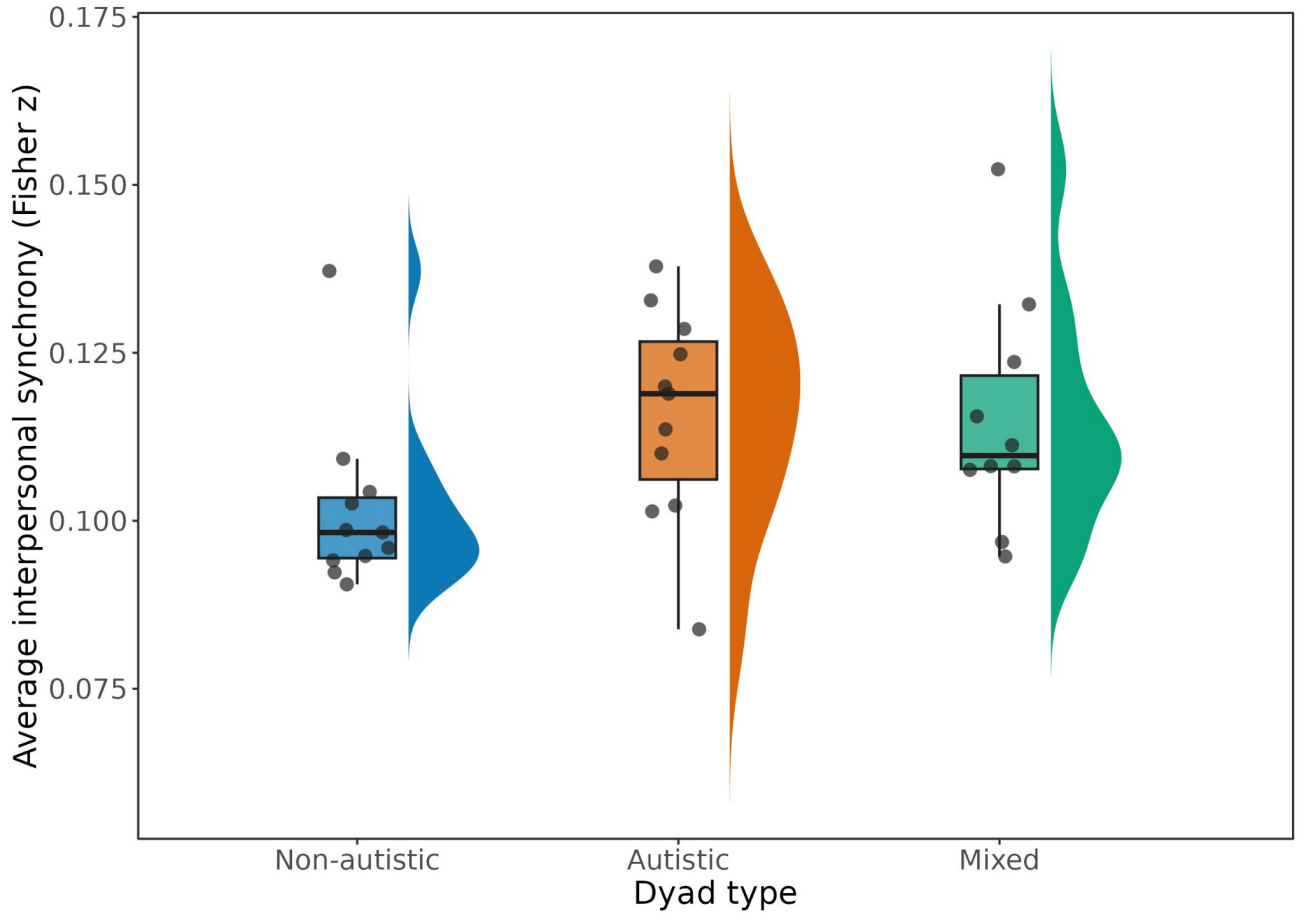
Average interpersonal synchrony by dyad type. Points represent dyads. Boxplots show medians and interquartile ranges, whiskers extend to 1.5 times the interquartile range, and half violins show the distribution within each dyad type. Interpersonal synchrony is plotted as the dyad-level mean Fisher *z*-transformed synchrony score across the task.

Estimated marginal means of interpersonal synchronization (Fisher z) from the dyad-window mixed model were 0.097 (SE = 0.004) for non-autistic dyads, 0.112 (SE = 0.004) for autistic dyads, and 0.107 (SE = 0.004) for mixed dyads (Figure 3). Tukey-adjusted pairwise contrasts showed higher interpersonal synchronization in autistic than in non-autistic dyads. For the non-autistic vs. autistic contrast, *b* = −0.015, 95% CI [−0.027, −0.002], *p* = 0.023. The remaining contrasts were not significant: non-autistic versus mixed, *b* = −0.010, 95% CI [−0.023, 0.003], *p* = 0.156; autistic vs. mixed, *b* = 0.004, 95% CI [−0.009, 0.018], *p* = 0.677 (see Supplemental Tables 5, 6). Thus, autistic dyads showed higher interpersonal synchronization than non-autistic dyads. Mixed dyads did not differ reliably from either group. The interval-adjusted and dyad-average analyses yielded substantively identical estimates (Supplemental Table 16). The minimum detectable effect analyses indicated that, with the present sample, pairwise interpersonal synchronization differences smaller than 0.58–0.59 standardized units would have been difficult to detect reliably (Supplemental Table 20).

### 3.3 Observed change in emotional self-regulation

Since the German version of the S-DERS has not yet been validated, Supplemental Table 15 reports the psychometric properties of the translated version.

For S-DERS Total, observed changes at average synchronization were small and non-significant in all three dyad types (non-autistic: *b* = 0.13, SE = 0.26, *p* = 0.618; autistic: *b* = −0.03, SE = 0.25, *p* = 0.888; mixed: *b* = −0.01, SE = 0.23, *p* = 0.973). Dyad-type contrasts in change were likewise nonsignificant (all ps >= 0.888).

For S-DERS Modulate, observed changes were also small (non-autistic: b = 0.15, SE = 0.26, p = 0.560; autistic: b = −0.17, SE = 0.24, *p* = 0.489; mixed: *b* = 0.06, SE = 0.23, *p* = 0.806). No dyad-type contrast in change reached significance (all ps >= 0.639). Baseline-adjusted post-task estimates led to the same substantive conclusion (all dyad-type estimate ps >= 0.531; all dyad-type contrast ps >= 0.734). Full marginal effects are reported in Supplemental Tables 11, 12.

### 3.4 Interpersonal synchronization and baseline-adjusted post-task emotional self-regulation

No within-dyad-type interpersonal synchronization slope reached significance for S-DERS Total (non-autistic: *b* = 0.06, *SE* = 0.14, *p* = 0.658; autistic: *b* = 0.10, *SE* = 0.14, *p* = 0.490; mixed: *b* = −0.35, *SE* = 0.20, *p* = 0.089). No pairwise difference between dyad types emerged (all *p*s >= 0.177).

**Figure 4:**
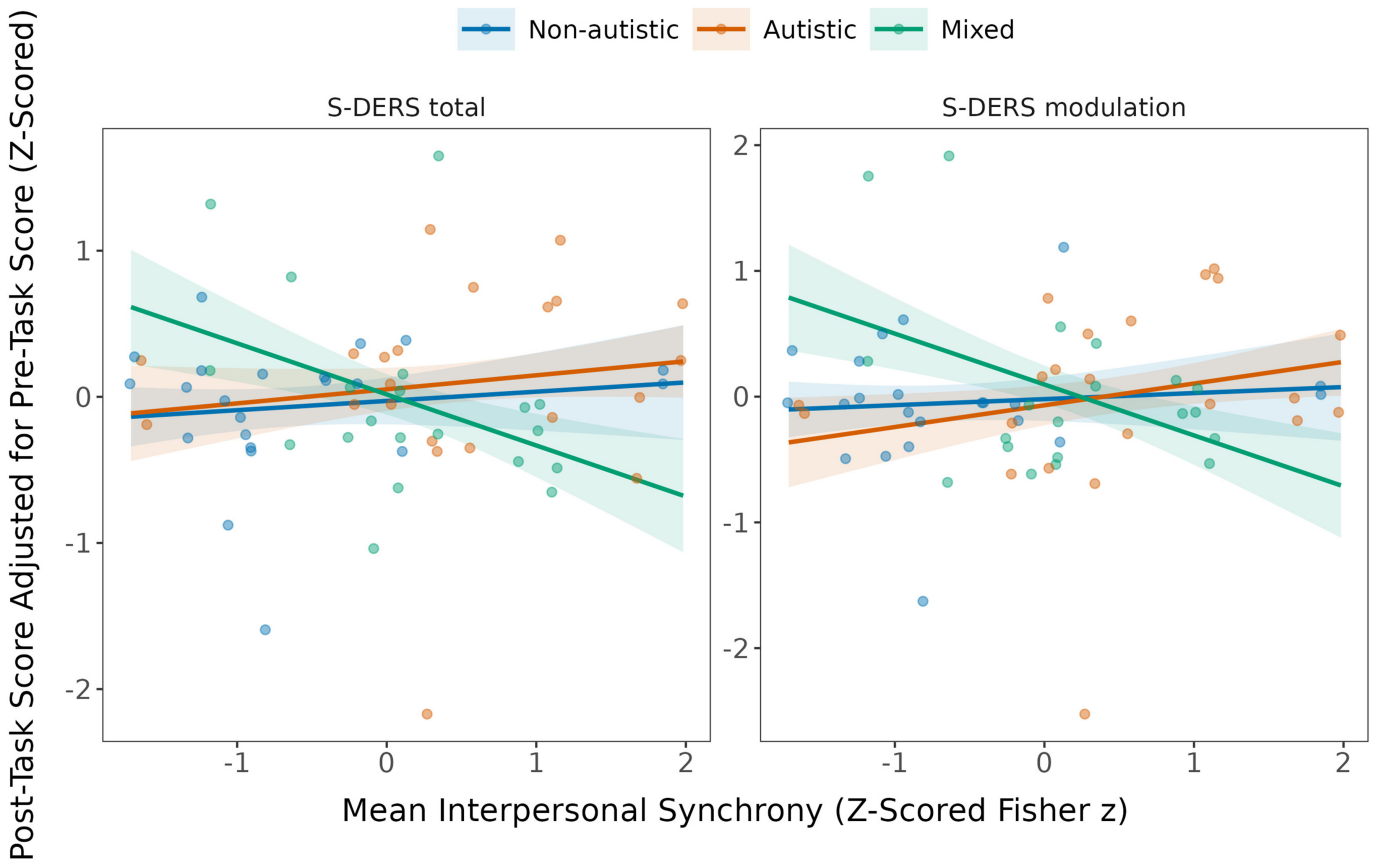
Association between mean interpersonal synchrony and baseline-adjusted post-task emotion regulation by dyad type. Lines show model-estimated slopes for the association between z-scored mean interpersonal synchrony and baseline-adjusted, standardized post-task S-DERS Total and S-DERS Modulate scores within each dyad type; shaded bands indicate plus or minus 1 standard error around the fitted values. Points show participant-level standardized post-task scores adjusted to the mean pre-task score. S-DERS = State Difficulties in Emotion Regulation Scale.

For S-DERS Modulate, the interpersonal synchronization slopes were likewise uncertain (non-autistic: *b* = 0.05, *SE* = 0.15, *p* = 0.758; autistic: *b* = 0.17, *SE* = 0.15, *p* = 0.258; mixed: *b* = −0.40, *SE* = 0.21, *p* = 0.067). No dyad-type contrast reached significance (all *p*s >= 0.086; Figure 4). The complementary observed change-score estimands again yielded the same substantive conclusion (all within-dyad-type slope *p*s >= 0.156; all dyad-type slope-contrast *p*s >= 0.223). Minimum detectable effect analyses indicated that within-dyad-type S-DERS-related interpersonal synchronization slopes smaller than 0.40–0.62 standardized post-task units per 1 SD of synchronization would have been difficult to identify with adequate power. The same was true of dyad differences in these slopes smaller than 0.58–0.76 standardized units. Full within-dyad slopes and pairwise contrasts are reported in Supplemental Tables 13, 14.

### 3.5 Observed change in affect

For positive affect, none of the dyad types showed a significant observed change at average interpersonal synchronization (non-autistic: *b* = 0.23, SE = 0.25, *p* = 0.359; autistic: *b* = −0.22, SE = 0.23, *p* = 0.346; mixed: *b* = −0.27, SE = 0.22, *p* = 0.238). Pairwise contrasts in change were likewise non-significant (all ps >= 0.307). For negative affect, observed changes were also small (non-autistic: *b* = −0.03, SE = 0.29, *p* = 0.925; autistic: *b* = 0.18, SE = 0.27, *p* = 0.503; mixed: *0* = −0.06, SE = 0.25, *p* = 0.808). No dyad-type contrast in change reached significance (all ps >= 0.788). Baseline-adjusted post-task estimates led to the same substantive conclusion (all dyad-type estimate ps >= 0.188; all dyad-type contrast ps >= 0.259). Full marginal effects are reported in Supplemental Tables 7, 8.

The results were unchanged when alexithymia was added to the interpersonal models (Supplemental Tables 21–25).

### 3.6 Interpersonal synchronization and baseline-adjusted post-task affect

We next examined whether mean interpersonal synchronization was associated with adjusted post-task affect within each dyad type. For positive affect, no interpersonal synchronization slope reached significance (non-autistic: *b* = −0.21, *SE* = 0.15, *p* = 0.170; autistic: *b* = 0.04, *SE* = 0.15, *p* = 0.810; mixed: *b* = 0.39, *SE* = 0.21, *p* = 0.082). The dyad differences among these slopes were also nonsignificant (all *p*s >= 0.076).

**Figure 5:**
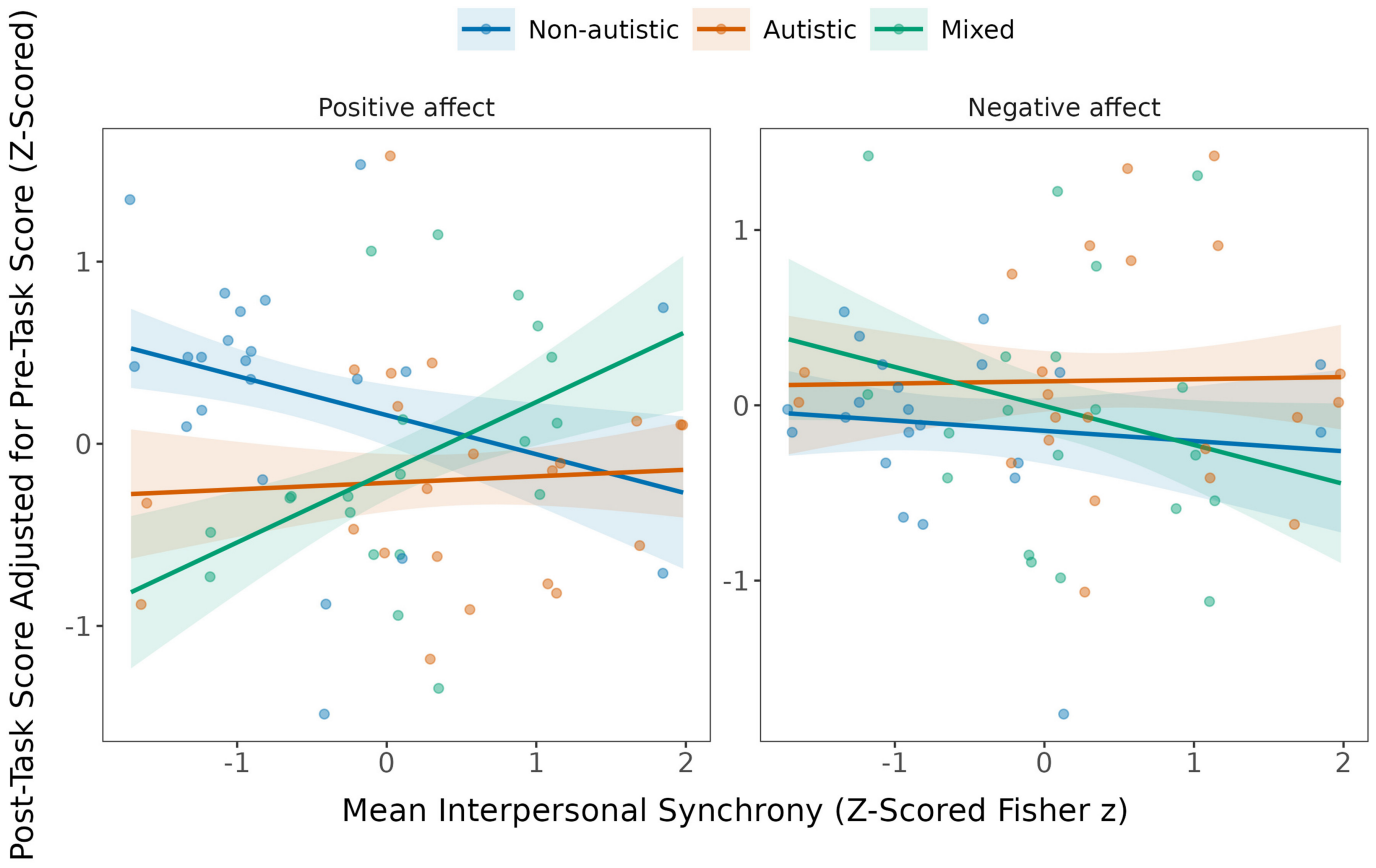
Association between mean interpersonal synchrony and baseline-adjusted post-task affect by dyad type. Lines show model-estimated slopes for the association between *z*-scored mean interpersonal synchrony and baseline-adjusted, standardized post-task positive and negative affect scores within each dyad type; shaded bands indicate plus or minus 1 standard error around the fitted values. Points show participant-level standardized post-task affect scores adjusted to the mean pre-task score.

For negative affect, no interpersonal synchronization slope reached significance (all *p*s >= 0.346). No pairwise difference between dyad types emerged (all *p*s >= 0.697; Figure 5). The complementary observed change-score estimands led to the same substantive conclusion (all within-dyad-type slope *p*s >= 0.209; all dyad-type slope-contrast *p*s >= 0.194). Minimum detectable effect analyses indicated that, with the present sample, within-dyad-type affect-related interpersonal synchronization slopes smaller than 0.43–0.67 standardized post-task units per 1 SD of synchronization would have been difficult to identify with adequate power. The same was true of dyad differences in these slopes smaller than 0.62–0.84 standardized units.

Across all interpersonal synchronization, affect, and S-DERS estimands, the practical-equivalence tests did not support equivalence under the specified ± 0.10 standardized bounds (Supplemental Table 19). Full within-dyad slopes and pairwise contrasts are reported in Supplemental Tables 9, 10.

## 4 Discussion

### 4.1 Interpersonal synchronization in different dyads

Contrary to our hypothesis and several previous findings, the autistic dyads in the present study exhibited stronger interpersonal synchronization during a non-verbal communication task than the non-autistic dyads. To our knowledge, only one other study has reported stronger interpersonal synchronization in autistic individuals (Glass and Yuill, 2024a). In a cooperative image-sorting task on two interconnected tablets, autistic children exhibited stronger interpersonal synchronization with each other than the non-autistic control group. Nevertheless, our finding appears counterintuitive in light of previous research suggesting reduced (Georgescu et al., 2020) or comparable levels of interpersonal synchronization (Mcnaughton et al., 2024; Efthimiou et al., 2025) in the autistic dyad. Our results revealed no group difference involving mixed dyads. However, our small sample and the inconclusive equivalence tests limit the robustness and generalizability of the results. Consequently, the observed group difference should be interpreted with caution until replicated in larger samples. Collecting data from autistic and mixed dyads is challenging, as was also the case in other studies of interpersonal synchronization involving dyads with one or two autistic individuals (Georgescu et al., 2020; Efthimiou et al., 2025; Mcnaughton et al., 2024). It may be most appropriate to conduct and publish smaller individual studies that can later be integrated into a meta-analysis.

A useful framework for interpreting our results is the Interactional Heterogeneity Hypothesis (Georgescu et al., 2020), which posits that social interaction is not a uniform ability but rather emerges from the fit between interaction partners' cognitive, perceptual, and communicative styles. According to this view, differences in interactional outcomes are not solely attributable to individual traits but arise from the degree of compatibility between partners during real-time coordination. To date, two studies of adults have examined interpersonal synchronization in autistic, mixed, and non-autistic dyads, with contradictory findings. Georgescu et al. (2020) examined interpersonal synchronization in several structured conversation tasks and found stronger interpersonal synchronization in non-autistic dyads than in the other two dyad types. A more recent study failed to confirm this group difference (Efthimiou et al., 2025). The authors explained this discrepancy by noting that the participants in their study completed only one 5-min conversational task, which was unstructured (participants were asked to have a conversation with one another). As a result, shared goals in the conversation may have been lacking, and the absence of multiple conversational situations may have prevented the emergence of cumulative effects through repeated exposure. In our study, the dyads had an average interpersonal synchronization of 0.105 (non-autistic dyads: 0.097; autistic dyads: 0.112; and mixed dyads: 0.107). In the study by Georgescu et al. (2020), the dyads had an average interpersonal synchronization of 0.09 (depending on the subtask, the values for the autistic dyad were between 0.071 and 0.114, for the mixed dyad between 0.74 and 0.99, and for the non-autistic dyad between 0.094 and 0.124). In Efthimiou et al. (2025) all three dyad types had an interpersonal synchronization of 0.16. Both the study task and the method used to measure interpersonal synchronization differed across the three studies, making direct comparison difficult. Interpersonal synchronization in our study was lower than that reported by Efthimiou et al. (2025) and higher than that reported by Georgescu et al. (2020).

When adolescents watched a video together for 2–5 min, non-autistic dyads exhibited significantly lower smile synchronization than mixed dyads (Mcnaughton et al., 2024). Comparisons involving the autistic dyad revealed no significant group differences relative to either mixed or non-autistic dyads. Autistic dyads showed slightly stronger interpersonal synchronization when controlling for the overall frequency of smiling. The experimental design did not allow us to determine conclusively whether the coordination of smiling between interaction partners reflected interpersonal synchronization in response to the shared visual stimulus or interpersonal synchronization directed toward one another.

Although participants completed only a 5-min whole-body movement task, it appeared to be sufficient to elicit stronger interpersonal synchronization in the autistic dyad than in the non-autistic dyad. Our results align with emerging empirical evidence indicating that autistic individuals can interact successfully with one another (Crompton et al., 2020b,c). However, the present findings did not confirm the Interactional Heterogeneity Hypothesis. Under this hypothesis, mixed dyads would show poorer interpersonal synchronization than the other two dyad types. This pattern did not emerge. In particular, the absence of a difference in interpersonal synchronization between non-autistic and mixed dyads contrasts with a number of previous studies (Mcnaughton et al., 2024; Georgescu et al., 2020). Given the relatively small sample size, the absence of significant differences between mixed and non-autistic dyads should be interpreted cautiously. Small-to-moderate effects may have remained undetected due to limited statistical power.

What factors might account for the observed group difference between autistic and non-autistic dyads? One possible explanation is that interpersonal synchronization is strongly dependent on task demands (Glass and Yuill, 2024b). Our study design diverges from the majority of prior research investigating interpersonal synchronization in autistic individuals (Georgescu et al., 2020; Glass and Yuill, 2024b; Carnevali et al., 2024; Mcnaughton et al., 2024). A key difference is that whole-body interpersonal synchronization usually played a minor role in previous research because the tasks involved conversation. In contrast, we used an alternative approach that recorded interpersonal synchronization while participants moved freely within a specified radius. This approach allowed us to analyze whole-body movements. The present study employed a non-verbal communication paradigm. This paradigm reduced reliance on language and familiarity with social tasks, factors that may have facilitated interpersonal synchronization among non-autistic individuals in studies using conversational contexts. Autistic individuals often experience greater difficulties in conversational situations. We did not systematically document how participants experienced the task. Nevertheless, limited familiarity with its non-conversational format may have reduced interpersonal synchronization performance among non-autistic individuals. Instead, successful task performance depended primarily on the continuous monitoring and adaptation of a partner's behavior.

In addition, conversational situations used in other interpersonal synchronization studies require a high degree of eye contact, which autistic people often struggle to maintain (Tanaka and Sung, 2016). In the BCT, however, the focus is less on the face and more on the entire body. To synchronize with someone else, people must have access to the other person's bodily movements, with which they synchronize (Miyata et al., 2017; Oullier et al., 2008). Reduced attention to the interaction partner weakens interpersonal synchronization (Bowsher-Murray et al., 2022). We did not use eye tracking to investigate this point specifically. Nevertheless, the focus on the whole body rather than the eyes may have facilitated interpersonal synchronization between autistic participants and their partners, at least compared with other studies.

However, these explanations can only account for why, in contrast to a number of previous studies, we did not observe higher interpersonal synchronization in the non-autistic dyads compared to the autistic dyads. To our knowledge, only one previous study has reported stronger interpersonal synchronization in autistic than in non-autistic dyads, and this effect was limited to a specific task condition (Glass and Yuill, 2024a). In that study, dyads consisting of autistic or non-autistic children completed two collaborative tasks: one involved a sorting activity performed jointly on a single tablet, whereas the other was conducted using two interconnected tablets. While no group differences were observed during the first task, autistic dyads exhibited higher interpersonal synchronization during the second task condition. The authors attributed this finding to the tendency of non-autistic dyads to engage more independently with their own tablet and to physically orient the device away from their interaction partner, thereby reducing opportunities to respond to the partner's behavior. This pattern was less pronounced in autistic dyads, possibly because tablet-based activities are frequently used in educational and therapeutic settings for skill development and practice (Kim and Clarke, 2015; Xin and Leonard, 2015). However, such an explanation cannot readily be applied to our study. Stronger interpersonal synchronization in autistic dyads compared to non-autistic dyads contradicts the findings of most previous studies. Whole-body interpersonal synchronization in individuals with autism spectrum condition has also received little empirical attention. Further research with larger samples is needed to better contextualize and interpret our findings.

### 4.2 Influence of synchronization on emotional self-regulation and on affect

The present findings indicate that the task did not lead to reliable observed changes in emotional self-regulation or in positive or negative affect across any of the three dyad types. In the baseline-adjusted models, post-task affect and emotional self-regulation were not predicted by the degree of interpersonal synchronization. The relationships between interpersonal synchronization and the post-task outcomes also did not differ between autistic, non-autistic, and mixed dyads. Given the relatively small sample size, it remains difficult to determine whether these findings reflect true null effects or are attributable to insufficient statistical power.

Consistent with the findings of Galbusera et al. (2019), we observed no effect of interpersonal synchronization on emotional self-regulation in non-autistic dyads based on the total S-DERS score. However, in contrast to the original study, we also found no improvement in emotional self-regulation on the modulate subscale of the S-DERS. A likely explanation for this discrepancy is the substantially larger sample in the reference study, which may have provided greater statistical power to detect small effects.

Similarly, our findings for affect, particularly positive affect, are inconsistent with a substantial body of literature linking interpersonal synchronization to positive interaction outcomes, including social connectedness, interaction enjoyment, cooperation, and positive affect (Galbusera et al., 2019; Mogan et al., 2017; Vicaria and Dickens, 2016). Given the discrepancy between our findings and previous research, we interpret the absence of significant effects of interpersonal synchronization on emotional self-regulation or affect primarily as a consequence of insufficient statistical power rather than as evidence that such relationships are absent. Future studies employing larger samples are therefore needed to examine these associations in greater detail.

## 5 Limitations

The primary limitation is the sample size. This is a common challenge in dyadic research (Paxton and Dale, 2013), as the dyad rather than the individual constitutes the unit of analysis, effectively reducing the sample size by half. This issue becomes even more pronounced when three different dyad types are investigated. Nevertheless, the present findings should be interpreted with caution and ideally replicated in larger samples.

## 6 Conclusion

Contrary to our initial hypothesis, autistic dyads exhibited higher levels of interpersonal synchronization than non-autistic dyads, while mixed dyads did not differ significantly from either group. Our findings extend previous research on interpersonal synchronization in autism spectrum condition by demonstrating that synchronization patterns may vary substantially depending on the interactional context and task demands. In particular, the use of a whole-body, non-verbal communication paradigm appears to reveal interactional dynamics that are not readily observable in conventional conversational settings. Future research should therefore investigate interpersonal synchronization across a broader range of embodied and naturalistic social contexts, such as by comparing whole-body movements with conversational interactions.

## Data Availability

The datasets presented in this study can be found in online repositories. The names of the repository/repositories and accession number(s) can be found in the article/Supplementary material.
