# Supplementary material for: Whole-body synchronization in autistic adults
Source: Front Hum Neurosci. 2026 Jul 31;20:1866968. doi: 10.3389/fnhum.2026.1866968 (PMC13473257; doi:10.3389/fnhum.2026.1866968)
Supplement: Supplementary file 1 [file Data_Sheet_1.docx]

# 1. Supplemental Tables for Interpersonal Synchrony and Questionnaire Analyses

## 1.1. Primary Model Coefficients

**Table 1**

*Estimated Coefficients for the Interpersonal Synchrony Model*

|  | | | *95% CI* | |  |
| --- | --- | --- | --- | --- | --- |
| Effect | Estimate | *SE* | *LL* | *UL* | *p* |
| **Fixed effects** |  |  |  |  |  |
| Intercept | .10*** | .004 | .09 | .10 | < .001 |
| Dyad Type: autistic | .01** | .005 | .004 | .03 | .009 |
| Dyad Type: mixed | .01 | .005 | -.001 | .02 | .067 |
| **Random effects** |  |  |  |  |  |
| Random intercept SD (Dyad Number) | .010 |  |  |  |  |
| Residual SD | .02 |  |  |  |  |
| **Model fit** |  |  |  |  |  |
| Observations | 321 |  |  |  |  |
| AIC | -1432.37 |  |  |  |  |
| BIC | -1413.52 |  |  |  |  |

*Note.* CI = confidence interval; LL = lower limit; UL = upper limit; SD = standard deviation. Fixed-effect estimates are reported relative to the non-autistic dyad group. Random-effect rows report estimated standard deviations. Model fit rows report the number of observations, Akaike information criterion (AIC), and Bayesian information criterion (BIC).

* p < .05. ** p < .01. *** p < .001.

**Table 2**

*Estimated Coefficients for the Baseline-Adjusted PANAS Models*

|  | | | *95% CI* | |  |
| --- | --- | --- | --- | --- | --- |
| Effect | Estimate | *SE* | *LL* | *UL* | *p* |
| ***Positive Affect*** |  |  |  |  |  |
| **Fixed effects** |  |  |  |  |  |
| Intercept | .16 | .17 | -.18 | .49 | .352 |
| Pre-Task Score (z) | .68*** | .09 | .51 | .86 | < .001 |
| Dyad Type: autistic | -.37 | .23 | -.83 | .09 | .112 |
| Dyad Type: mixed | -.31 | .23 | -.76 | .14 | .169 |
| Interpersonal Synchrony Slope: non-autistic | -.21 | .15 | -.52 | .09 | .163 |
| Interpersonal Synchrony Slope: autistic | .04 | .15 | -.26 | .33 | .809 |
| Interpersonal Synchrony Slope: mixed | .39 | .21 | -.04 | .81 | .076 |
| **Random effects** |  |  |  |  |  |
| Random intercept SD (Dyad Number) | .00 |  |  |  |  |
| Residual SD | .65 |  |  |  |  |
| **Model fit** |  |  |  |  |  |
| Observations | 64 |  |  |  |  |
| AIC | 152.83 |  |  |  |  |
| BIC | 172.26 |  |  |  |  |
| ***Negative Affect*** |  |  |  |  |  |
| **Fixed effects** |  |  |  |  |  |
| Intercept | -.15 | .19 | -.53 | .24 | .444 |
| Pre-Task Score (z) | .74*** | .08 | .58 | .91 | < .001 |
| Dyad Type: autistic | .28 | .26 | -.24 | .81 | .279 |
| Dyad Type: mixed | .14 | .25 | -.37 | .66 | .576 |
| Interpersonal Synchrony Slope: non-autistic | -.06 | .17 | -.40 | .29 | .732 |
| Interpersonal Synchrony Slope: autistic | .01 | .17 | -.33 | .36 | .942 |
| Interpersonal Synchrony Slope: mixed | -.22 | .23 | -.70 | .25 | .346 |
| **Random effects** |  |  |  |  |  |
| Random intercept SD (Dyad Number) | .31 |  |  |  |  |
| Residual SD | .58 |  |  |  |  |
| **Model fit** |  |  |  |  |  |
| Observations | 64 |  |  |  |  |
| AIC | 150.30 |  |  |  |  |
| BIC | 169.73 |  |  |  |  |

*Note.* CI = confidence interval; LL = lower limit; UL = upper limit; SD = standard deviation. Fixed-effect estimates are reported relative to the non-autistic dyad group, with post-task scores adjusted for the corresponding pre-task score. Random-effect rows report estimated standard deviations. Model fit rows report the number of observations, Akaike information criterion (AIC), and Bayesian information criterion (BIC).

* p < .05. ** p < .01. *** p < .001.

**Table 3**

*Estimated Coefficients for the Baseline-Adjusted S-DERS Models*

|  | | | *95% CI* | |  |
| --- | --- | --- | --- | --- | --- |
| Effect | Estimate | *SE* | *LL* | *UL* | *p* |
| ***S-DERS Total*** |  |  |  |  |  |
| **Fixed effects** |  |  |  |  |  |
| Intercept | -.03 | .16 | -.35 | .29 | .862 |
| Pre-Task Score (z) | .75*** | .08 | .59 | .92 | < .001 |
| Dyad Type: autistic | .08 | .22 | -.36 | .52 | .722 |
| Dyad Type: mixed | .04 | .21 | -.38 | .47 | .835 |
| Interpersonal Synchrony Slope: non-autistic | .06 | .14 | -.22 | .35 | .656 |
| Interpersonal Synchrony Slope: autistic | .10 | .14 | -.18 | .37 | .487 |
| Interpersonal Synchrony Slope: mixed | -.35 | .20 | -.74 | .05 | .082 |
| **Random effects** |  |  |  |  |  |
| Random intercept SD (Dyad Number) | .00 |  |  |  |  |
| Residual SD | .61 |  |  |  |  |
| **Model fit** |  |  |  |  |  |
| Observations | 64 |  |  |  |  |
| AIC | 144.36 |  |  |  |  |
| BIC | 163.79 |  |  |  |  |
| ***S-DERS Modulate*** |  |  |  |  |  |
| **Fixed effects** |  |  |  |  |  |
| Intercept | -.02 | .17 | -.37 | .33 | .910 |
| Pre-Task Score (z) | .73*** | .09 | .56 | .90 | < .001 |
| Dyad Type: autistic | -.05 | .24 | -.52 | .43 | .837 |
| Dyad Type: mixed | .12 | .23 | -.35 | .58 | .619 |
| Interpersonal Synchrony Slope: non-autistic | .05 | .15 | -.26 | .36 | .756 |
| Interpersonal Synchrony Slope: autistic | .17 | .15 | -.13 | .47 | .252 |
| Interpersonal Synchrony Slope: mixed | -.40 | .21 | -.83 | .02 | .061 |
| **Random effects** |  |  |  |  |  |
| Random intercept SD (Dyad Number) | .00 |  |  |  |  |
| Residual SD | .66 |  |  |  |  |
| **Model fit** |  |  |  |  |  |
| Observations | 64 |  |  |  |  |
| AIC | 154.35 |  |  |  |  |
| BIC | 173.78 |  |  |  |  |

*Note.* CI = confidence interval; LL = lower limit; UL = upper limit; SD = standard deviation. Fixed-effect estimates are reported relative to the non-autistic dyad group, with post-task scores adjusted for the corresponding pre-task score. Random-effect rows report estimated standard deviations. Model fit rows report the number of observations, Akaike information criterion (AIC), and Bayesian information criterion (BIC).

* p < .05. ** p < .01. *** p < .001.

## 1.2. Primary Marginal Estimates and Contrasts

**Table 4**

*Frequentist Model Fit Summary*

| Model | Observations | Marginal R2 | Conditional R2 | Singular |
| --- | --- | --- | --- | --- |
| Interpersonal Synchrony | 321 | 0.06 | 0.19 | No |
| Positive Affect | 64 | 0.59 | 0.59 | Yes |
| Negative Affect | 64 | 0.59 | 0.68 | No |
| S-DERS Total | 64 | 0.64 | 0.64 | Yes |
| S-DERS Modulate | 64 | 0.58 | 0.58 | Yes |

*Note.* Marginal R2 = variance explained by fixed effects alone. Conditional R2 = variance explained by fixed and random effects combined. Singular = boundary fit warning from the fitted mixed model.

**Table 5**

*Estimated Marginal Means for Interpersonal Synchrony by Dyad Type*

| dyad | estimate | SE | 95% CI | t | p |
| --- | --- | --- | --- | --- | --- |
| Non-autistic | 0.10*** | 0.004 | [0.09, 0.10] | 26.57 | < .001 |
| Autistic | 0.11*** | 0.004 | [0.10, 0.12] | 30.55 | < .001 |
| Mixed | 0.11*** | 0.004 | [0.10, 0.12] | 28.01 | < .001 |

*Note.* CI = confidence interval; SE = standard error. Estimates are estimated marginal means for interpersonal synchrony by dyad type.

* p < .05. ** p < .01. *** p < .001.

**Table 6**

*Contrasts for Interpersonal Synchrony by Dyad Type*

| contrast | estimate | SE | 95% CI | t | p |
| --- | --- | --- | --- | --- | --- |
| Non-autistic vs Autistic | -0.01* | 0.005 | [-0.03, -0.002] | -2.82 | .023 |
| Non-autistic vs Mixed | -0.01 | 0.005 | [-0.02, 0.003] | -1.90 | .156 |
| Autistic vs Mixed | 0.004 | 0.005 | [-0.009, 0.02] | 0.85 | .677 |

*Note.* CI = confidence interval; SE = standard error. Estimates are pairwise contrasts in interpersonal synchrony between levels of dyad type.

* p < .05. ** p < .01. *** p < .001.

**Table 7**

*Baseline-Adjusted PANAS Post-Task Scores by Dyad Type*

| outcome | dyad | estimate | SE | 95% CI | t | p |
| --- | --- | --- | --- | --- | --- | --- |
| Positive Affect | Non-autistic | 0.16 | 0.17 | [-0.19, 0.50] | 0.94 | .357 |
| Positive Affect | Autistic | -0.21 | 0.16 | [-0.54, 0.11] | -1.35 | .188 |
| Positive Affect | Mixed | -0.16 | 0.15 | [-0.46, 0.15] | -1.04 | .307 |
| Negative Affect | Non-autistic | -0.15 | 0.19 | [-0.53, 0.24] | -0.78 | .444 |
| Negative Affect | Autistic | 0.14 | 0.17 | [-0.22, 0.49] | 0.79 | .439 |
| Negative Affect | Mixed | -0.004 | 0.17 | [-0.35, 0.34] | -0.02 | .983 |

*Note.* Estimates are baseline-adjusted post-task PANAS scores at average interpersonal synchrony and average pre-task PANAS score.

**Table 8**

*Contrasts of Baseline-Adjusted PANAS Post-Task Scores by Dyad Type*

| outcome | contrast | estimate | SE | 95% CI | t | p |
| --- | --- | --- | --- | --- | --- | --- |
| Positive Affect | Non-autistic vs Autistic | 0.37 | 0.23 | [-0.20, 0.94] | 1.61 | .259 |
| Positive Affect | Non-autistic vs Mixed | 0.31 | 0.23 | [-0.25, 0.87] | 1.39 | .360 |
| Positive Affect | Autistic vs Mixed | -0.06 | 0.22 | [-0.60, 0.49] | -0.26 | .962 |
| Negative Affect | Non-autistic vs Autistic | -0.28 | 0.26 | [-0.92, 0.35] | -1.11 | .520 |
| Negative Affect | Non-autistic vs Mixed | -0.14 | 0.25 | [-0.77, 0.48] | -0.57 | .839 |
| Negative Affect | Autistic vs Mixed | 0.14 | 0.24 | [-0.46, 0.74] | 0.58 | .830 |

*Note.* Estimates are pairwise dyad-type contrasts in baseline-adjusted post-task PANAS scores.

**Table 9**

*Interpersonal Synchrony Slopes for Baseline-Adjusted PANAS Post-Task Scores by Dyad Type*

| outcome | dyad | estimate | SE | 95% CI | t | p |
| --- | --- | --- | --- | --- | --- | --- |
| Positive Affect | Non-autistic | -0.21 | 0.15 | [-0.53, 0.10] | -1.41 | .170 |
| Positive Affect | Autistic | 0.04 | 0.15 | [-0.27, 0.34] | 0.24 | .810 |
| Positive Affect | Mixed | 0.39 | 0.21 | [-0.05, 0.82] | 1.80 | .082 |
| Negative Affect | Non-autistic | -0.06 | 0.17 | [-0.40, 0.29] | -0.35 | .733 |
| Negative Affect | Autistic | 0.01 | 0.17 | [-0.33, 0.36] | 0.07 | .942 |
| Negative Affect | Mixed | -0.22 | 0.23 | [-0.70, 0.25] | -0.96 | .346 |

*Note.* Estimates are simple slopes relating interpersonal synchrony to baseline-adjusted post-task PANAS scores within each dyad type.

**Table 10**

*Contrasts of Interpersonal Synchrony Slopes for Baseline-Adjusted PANAS Post-Task Scores*

| outcome | contrast | estimate | SE | 95% CI | t | p |
| --- | --- | --- | --- | --- | --- | --- |
| Positive Affect | Non-autistic vs Autistic | -0.25 | 0.21 | [-0.78, 0.28] | -1.18 | .475 |
| Positive Affect | Non-autistic vs Mixed | -0.60 | 0.26 | [-1.25, 0.05] | -2.28 | .076 |
| Positive Affect | Autistic vs Mixed | -0.35 | 0.26 | [-0.99, 0.29] | -1.36 | .378 |
| Negative Affect | Non-autistic vs Autistic | -0.07 | 0.24 | [-0.66, 0.52] | -0.30 | .953 |
| Negative Affect | Non-autistic vs Mixed | 0.16 | 0.29 | [-0.55, 0.88] | 0.57 | .835 |
| Negative Affect | Autistic vs Mixed | 0.23 | 0.29 | [-0.48, 0.95] | 0.82 | .697 |

*Note.* Estimates are pairwise dyad-type contrasts in the simple slopes relating interpersonal synchrony to baseline-adjusted post-task PANAS scores.

**Table 11**

*Baseline-Adjusted S-DERS Post-Task Scores by Dyad Type*

| outcome | dyad | estimate | SE | 95% CI | t | p |
| --- | --- | --- | --- | --- | --- | --- |
| SDERS Total | Non-autistic | -0.03 | 0.16 | [-0.36, 0.30] | -0.17 | .863 |
| SDERS Total | Autistic | 0.05 | 0.15 | [-0.25, 0.35] | 0.35 | .731 |
| SDERS Total | Mixed | 0.02 | 0.14 | [-0.27, 0.30] | 0.12 | .905 |
| SDERS Modul | Non-autistic | -0.02 | 0.17 | [-0.38, 0.34] | -0.11 | .910 |
| SDERS Modul | Autistic | -0.07 | 0.16 | [-0.40, 0.26] | -0.43 | .670 |
| SDERS Modul | Mixed | 0.10 | 0.15 | [-0.22, 0.41] | 0.63 | .531 |

*Note.* Estimates are baseline-adjusted post-task S-DERS scores at average interpersonal synchrony and average pre-task S-DERS score.

**Table 12**

*Contrasts of Baseline-Adjusted S-DERS Post-Task Scores by Dyad Type*

| outcome | contrast | estimate | SE | 95% CI | t | p |
| --- | --- | --- | --- | --- | --- | --- |
| SDERS Total | Non-autistic vs Autistic | -0.08 | 0.22 | [-0.63, 0.47] | -0.36 | .932 |
| SDERS Total | Non-autistic vs Mixed | -0.04 | 0.21 | [-0.57, 0.49] | -0.21 | .976 |
| SDERS Total | Autistic vs Mixed | 0.03 | 0.20 | [-0.47, 0.54] | 0.17 | .984 |
| SDERS Modul | Non-autistic vs Autistic | 0.05 | 0.24 | [-0.54, 0.64] | 0.21 | .977 |
| SDERS Modul | Non-autistic vs Mixed | -0.12 | 0.23 | [-0.69, 0.46] | -0.50 | .872 |
| SDERS Modul | Autistic vs Mixed | -0.17 | 0.22 | [-0.71, 0.38] | -0.75 | .734 |

*Note.* Estimates are pairwise dyad-type contrasts in baseline-adjusted post-task S-DERS scores.

**Table 13**

*Interpersonal Synchrony Slopes for Baseline-Adjusted S-DERS Post-Task Scores by Dyad Type*

| outcome | dyad | estimate | SE | 95% CI | t | p |
| --- | --- | --- | --- | --- | --- | --- |
| SDERS Total | Non-autistic | 0.06 | 0.14 | [-0.23, 0.35] | 0.45 | .658 |
| SDERS Total | Autistic | 0.10 | 0.14 | [-0.19, 0.38] | 0.70 | .490 |
| SDERS Total | Mixed | -0.35 | 0.20 | [-0.76, 0.06] | -1.77 | .089 |
| SDERS Modul | Non-autistic | 0.05 | 0.15 | [-0.27, 0.36] | 0.31 | .758 |
| SDERS Modul | Autistic | 0.17 | 0.15 | [-0.13, 0.48] | 1.16 | .258 |
| SDERS Modul | Mixed | -0.40 | 0.21 | [-0.84, 0.03] | -1.91 | .067 |

*Note.* Estimates are simple slopes relating interpersonal synchrony to baseline-adjusted post-task S-DERS scores within each dyad type.

**Table 14**

*Contrasts of Interpersonal Synchrony Slopes for Baseline-Adjusted S-DERS Post-Task Scores*

| outcome | contrast | estimate | SE | 95% CI | t | p |
| --- | --- | --- | --- | --- | --- | --- |
| SDERS Total | Non-autistic vs Autistic | -0.03 | 0.20 | [-0.52, 0.46] | -0.17 | .985 |
| SDERS Total | Non-autistic vs Mixed | 0.41 | 0.24 | [-0.19, 1.01] | 1.71 | .220 |
| SDERS Total | Autistic vs Mixed | 0.45 | 0.24 | [-0.16, 1.05] | 1.84 | .177 |
| SDERS Modul | Non-autistic vs Autistic | -0.12 | 0.21 | [-0.66, 0.41] | -0.58 | .831 |
| SDERS Modul | Non-autistic vs Mixed | 0.45 | 0.26 | [-0.20, 1.10] | 1.73 | .212 |
| SDERS Modul | Autistic vs Mixed | 0.58 | 0.26 | [-0.07, 1.22] | 2.23 | .086 |

*Note.* Estimates are pairwise dyad-type contrasts in the simple slopes relating interpersonal synchrony to baseline-adjusted post-task S-DERS scores.

## 1.3. S-DERS Reliability

**Table 15**

*S-DERS Internal Reliability Summary*

| Timepoint | Scale | Items | n | Missing | Alpha | Std. Alpha | Average r | 95% CI |
| --- | --- | --- | --- | --- | --- | --- | --- | --- |
| Pre | S-DERS Total | 21 | 66 | 0 | 0.87 | 0.89 | 0.27 | [0.82, 0.91] |
| Pre | Nonacceptance | 7 | 66 | 0 | 0.86 | 0.88 | 0.50 | [0.80, 0.91] |
| Pre | Modulate | 7 | 66 | 0 | 0.84 | 0.85 | 0.45 | [0.77, 0.89] |
| Pre | Awareness | 5 | 66 | 0 | 0.58 | 0.58 | 0.22 | [0.39, 0.72] |
| Pre | Clarity | 2 | 66 | 0 | 0.77 | 0.78 | 0.64 | [0.63, 0.86] |
| Post | S-DERS Total | 21 | 65 | 1 | 0.88 | 0.90 | 0.30 | [0.84, 0.92] |
| Post | Nonacceptance | 7 | 66 | 0 | 0.91 | 0.92 | 0.63 | [0.87, 0.94] |
| Post | Modulate | 7 | 65 | 1 | 0.85 | 0.86 | 0.46 | [0.78, 0.90] |
| Post | Awareness | 5 | 66 | 0 | 0.74 | 0.74 | 0.37 | [0.63, 0.83] |
| Post | Clarity | 2 | 66 | 0 | 0.69 | 0.70 | 0.54 | [0.50, 0.81] |

*Note.* Alpha = Cronbach's alpha; Std. Alpha = standardized Cronbach's alpha; CI = confidence interval. Reliability estimates use the S-DERS item responses underlying the reported questionnaire analyses.

## 1.4. Supplemental Model Comparisons

**Table 16**

*Interval-Adjusted and Dyad-Average Synchrony Model Comparison*

| Estimand | Term | Comparison Model | Primary | Comparison | Delta / SE | SE Ratio |
| --- | --- | --- | --- | --- | --- | --- |
| Mean | Non-Autistic Dyads | Dyad and Interval Random Intercepts | 0.10 | 0.10 | 0.000000 | 1.00 |
| Mean | Autistic Dyads | Dyad and Interval Random Intercepts | 0.11 | 0.11 | 0.000000 | 1.00 |
| Mean | Mixed Dyads | Dyad and Interval Random Intercepts | 0.11 | 0.11 | 0.000000 | 1.00 |
| Dyad Contrast | Non-autistic vs Autistic | Dyad and Interval Random Intercepts | -0.01 | -0.01 | 0.000000 | 1.00 |
| Dyad Contrast | Non-autistic vs Mixed | Dyad and Interval Random Intercepts | -0.01 | -0.01 | 0.000000 | 1.00 |
| Dyad Contrast | Autistic vs Mixed | Dyad and Interval Random Intercepts | 0.004 | 0.004 | 0.000000 | 1.00 |
| Mean | Non-Autistic Dyads | Dyad-Average Model | 0.10 | 0.10 | 0.000000 | 1.00 |
| Mean | Autistic Dyads | Dyad-Average Model | 0.11 | 0.11 | 0.000000 | 1.00 |
| Mean | Mixed Dyads | Dyad-Average Model | 0.11 | 0.11 | 0.001 | 1.00 |
| Dyad Contrast | Non-autistic vs Autistic | Dyad-Average Model | -0.01 | -0.01 | 0.000000 | 1.00 |
| Dyad Contrast | Non-autistic vs Mixed | Dyad-Average Model | -0.01 | -0.01 | 0.0004 | 1.00 |
| Dyad Contrast | Autistic vs Mixed | Dyad-Average Model | 0.004 | 0.004 | 0.0004 | 1.00 |

*Note.* Comparison estimates are evaluated against the primary dyad random-intercept interval-level synchrony model. No comparison changed the conventional p < .05 decision status.

**Table 17**

*Observed Change-Score Model Comparison*

| Outcome | Estimand | Term | Primary | Comparison | Delta / SE | SE Ratio |
| --- | --- | --- | --- | --- | --- | --- |
| Positive Affect | Adjusted Post-Task Score | Non-Autistic Dyads | 0.16 | 0.23 | 0.44 | 1.48 |
| Positive Affect | Adjusted Post-Task Score | Autistic Dyads | -0.21 | -0.22 | 0.05 | 1.46 |
| Positive Affect | Adjusted Post-Task Score | Mixed Dyads | -0.16 | -0.27 | 0.74 | 1.47 |
| Positive Affect | Dyad Contrast: Adjusted Score | Non-autistic vs Autistic | 0.37 | 0.45 | 0.35 | 1.47 |
| Positive Affect | Dyad Contrast: Adjusted Score | Non-autistic vs Mixed | 0.31 | 0.50 | 0.82 | 1.48 |
| Positive Affect | Dyad Contrast: Adjusted Score | Autistic vs Mixed | -0.06 | 0.04 | 0.47 | 1.46 |
| Positive Affect | Synchrony Slope | Non-Autistic Dyads | -0.21 | -0.29 | 0.49 | 1.48 |
| Positive Affect | Synchrony Slope | Autistic Dyads | 0.04 | 0.010 | 0.18 | 1.47 |
| Positive Affect | Synchrony Slope | Mixed Dyads | 0.39 | 0.39 | 0.03 | 1.44 |
| Positive Affect | Dyad Contrast: Slope | Non-autistic vs Autistic | -0.25 | -0.30 | 0.23 | 1.47 |
| Positive Affect | Dyad Contrast: Slope | Non-autistic vs Mixed | -0.60 | -0.68 | 0.31 | 1.45 |
| Positive Affect | Dyad Contrast: Slope | Autistic vs Mixed | -0.35 | -0.38 | 0.12 | 1.46 |
| Negative Affect | Adjusted Post-Task Score | Non-Autistic Dyads | -0.15 | -0.03 | 0.63 | 1.52 |
| Negative Affect | Adjusted Post-Task Score | Autistic Dyads | 0.14 | 0.18 | 0.26 | 1.54 |
| Negative Affect | Adjusted Post-Task Score | Mixed Dyads | -0.004 | -0.06 | 0.36 | 1.53 |
| Negative Affect | Dyad Contrast: Adjusted Score | Non-autistic vs Autistic | -0.28 | -0.21 | 0.29 | 1.53 |
| Negative Affect | Dyad Contrast: Adjusted Score | Non-autistic vs Mixed | -0.14 | 0.04 | 0.71 | 1.52 |
| Negative Affect | Dyad Contrast: Adjusted Score | Autistic vs Mixed | 0.14 | 0.24 | 0.43 | 1.53 |
| Negative Affect | Synchrony Slope | Non-Autistic Dyads | -0.06 | -0.07 | 0.06 | 1.54 |
| Negative Affect | Synchrony Slope | Autistic Dyads | 0.01 | -0.24 | 1.52 | 1.50 |
| Negative Affect | Synchrony Slope | Mixed Dyads | -0.22 | -0.17 | 0.23 | 1.53 |
| Negative Affect | Dyad Contrast: Slope | Non-autistic vs Autistic | -0.07 | 0.17 | 1.03 | 1.52 |
| Negative Affect | Dyad Contrast: Slope | Non-autistic vs Mixed | 0.16 | 0.10 | 0.22 | 1.53 |
| Negative Affect | Dyad Contrast: Slope | Autistic vs Mixed | 0.23 | -0.07 | 1.07 | 1.51 |
| S-DERS Total | Adjusted Post-Task Score | Non-Autistic Dyads | -0.03 | 0.13 | 1.00 | 1.64 |
| S-DERS Total | Adjusted Post-Task Score | Autistic Dyads | 0.05 | -0.03 | 0.58 | 1.66 |
| S-DERS Total | Adjusted Post-Task Score | Mixed Dyads | 0.02 | -0.008 | 0.18 | 1.68 |
| S-DERS Total | Dyad Contrast: Adjusted Score | Non-autistic vs Autistic | -0.08 | 0.17 | 1.11 | 1.62 |
| S-DERS Total | Dyad Contrast: Adjusted Score | Non-autistic vs Mixed | -0.04 | 0.14 | 0.87 | 1.65 |
| S-DERS Total | Dyad Contrast: Adjusted Score | Autistic vs Mixed | 0.03 | -0.03 | 0.31 | 1.68 |
| S-DERS Total | Synchrony Slope | Non-Autistic Dyads | 0.06 | 0.15 | 0.62 | 1.68 |
| S-DERS Total | Synchrony Slope | Autistic Dyads | 0.10 | 0.10 | 0.04 | 1.68 |
| S-DERS Total | Synchrony Slope | Mixed Dyads | -0.35 | -0.36 | 0.07 | 1.65 |
| S-DERS Total | Dyad Contrast: Slope | Non-autistic vs Autistic | -0.03 | 0.05 | 0.41 | 1.68 |
| S-DERS Total | Dyad Contrast: Slope | Non-autistic vs Mixed | 0.41 | 0.51 | 0.42 | 1.67 |
| S-DERS Total | Dyad Contrast: Slope | Autistic vs Mixed | 0.45 | 0.47 | 0.08 | 1.65 |
| S-DERS Modulate | Adjusted Post-Task Score | Non-Autistic Dyads | -0.02 | 0.15 | 1.00 | 1.50 |
| S-DERS Modulate | Adjusted Post-Task Score | Autistic Dyads | -0.07 | -0.17 | 0.64 | 1.52 |
| S-DERS Modulate | Adjusted Post-Task Score | Mixed Dyads | 0.10 | 0.06 | 0.26 | 1.52 |
| S-DERS Modulate | Dyad Contrast: Adjusted Score | Non-autistic vs Autistic | 0.05 | 0.32 | 1.15 | 1.49 |
| S-DERS Modulate | Dyad Contrast: Adjusted Score | Non-autistic vs Mixed | -0.12 | 0.10 | 0.91 | 1.49 |
| S-DERS Modulate | Dyad Contrast: Adjusted Score | Autistic vs Mixed | -0.17 | -0.23 | 0.28 | 1.53 |
| S-DERS Modulate | Synchrony Slope | Non-Autistic Dyads | 0.05 | 0.12 | 0.44 | 1.52 |
| S-DERS Modulate | Synchrony Slope | Autistic Dyads | 0.17 | 0.20 | 0.20 | 1.53 |
| S-DERS Modulate | Synchrony Slope | Mixed Dyads | -0.40 | -0.47 | 0.31 | 1.52 |
| S-DERS Modulate | Dyad Contrast: Slope | Non-autistic vs Autistic | -0.12 | -0.09 | 0.18 | 1.52 |
| S-DERS Modulate | Dyad Contrast: Slope | Non-autistic vs Mixed | 0.45 | 0.59 | 0.51 | 1.53 |
| S-DERS Modulate | Dyad Contrast: Slope | Autistic vs Mixed | 0.58 | 0.67 | 0.37 | 1.52 |

*Note.* Comparison estimates are from observed change-score models and are evaluated against the primary baseline-adjusted post-task questionnaire models. No comparison changed the conventional p < .05 decision status.

**Table 18**

*Simplified Random-Effect Model Comparison*

| Outcome | Estimand | Term | Primary | Comparison | Delta / SE | SE Ratio |
| --- | --- | --- | --- | --- | --- | --- |
| Positive Affect | Adjusted Post-Task Score | Non-Autistic Dyads | 0.16 | 0.16 | 0.000000 | 1.00 |
| Positive Affect | Adjusted Post-Task Score | Autistic Dyads | -0.21 | -0.21 | 0.000000 | 1.00 |
| Positive Affect | Adjusted Post-Task Score | Mixed Dyads | -0.16 | -0.16 | 0.000000 | 1.00 |
| Positive Affect | Dyad Contrast: Adjusted Score | Non-autistic vs Autistic | 0.37 | 0.37 | 0.000000 | 1.00 |
| Positive Affect | Dyad Contrast: Adjusted Score | Non-autistic vs Mixed | 0.31 | 0.31 | 0.000000 | 1.00 |
| Positive Affect | Dyad Contrast: Adjusted Score | Autistic vs Mixed | -0.06 | -0.06 | 0.000000 | 1.00 |
| Positive Affect | Synchrony Slope | Non-Autistic Dyads | -0.21 | -0.21 | 0.000000 | 1.00 |
| Positive Affect | Synchrony Slope | Autistic Dyads | 0.04 | 0.04 | 0.000000 | 1.00 |
| Positive Affect | Synchrony Slope | Mixed Dyads | 0.39 | 0.39 | 0.000000 | 1.00 |
| Positive Affect | Dyad Contrast: Slope | Non-autistic vs Autistic | -0.25 | -0.25 | 0.00 | 1.00 |
| Positive Affect | Dyad Contrast: Slope | Non-autistic vs Mixed | -0.60 | -0.60 | 0.000000 | 1.00 |
| Positive Affect | Dyad Contrast: Slope | Autistic vs Mixed | -0.35 | -0.35 | 0.000000 | 1.00 |
| S-DERS Total | Adjusted Post-Task Score | Non-Autistic Dyads | -0.03 | -0.03 | 0.000000 | 1.00 |
| S-DERS Total | Adjusted Post-Task Score | Autistic Dyads | 0.05 | 0.05 | 0.000000 | 1.00 |
| S-DERS Total | Adjusted Post-Task Score | Mixed Dyads | 0.02 | 0.02 | 0.000000 | 1.00 |
| S-DERS Total | Dyad Contrast: Adjusted Score | Non-autistic vs Autistic | -0.08 | -0.08 | 0.000000 | 1.00 |
| S-DERS Total | Dyad Contrast: Adjusted Score | Non-autistic vs Mixed | -0.04 | -0.04 | 0.000000 | 1.00 |
| S-DERS Total | Dyad Contrast: Adjusted Score | Autistic vs Mixed | 0.03 | 0.03 | 0.000000 | 1.00 |
| S-DERS Total | Synchrony Slope | Non-Autistic Dyads | 0.06 | 0.06 | 0.000000 | 1.00 |
| S-DERS Total | Synchrony Slope | Autistic Dyads | 0.10 | 0.10 | 0.000000 | 1.00 |
| S-DERS Total | Synchrony Slope | Mixed Dyads | -0.35 | -0.35 | 0.000000 | 1.00 |
| S-DERS Total | Dyad Contrast: Slope | Non-autistic vs Autistic | -0.03 | -0.03 | 0.00 | 1.00 |
| S-DERS Total | Dyad Contrast: Slope | Non-autistic vs Mixed | 0.41 | 0.41 | 0.000000 | 1.00 |
| S-DERS Total | Dyad Contrast: Slope | Autistic vs Mixed | 0.45 | 0.45 | 0.00 | 1.00 |
| S-DERS Modulate | Adjusted Post-Task Score | Non-Autistic Dyads | -0.02 | -0.02 | 0.000000 | 1.00 |
| S-DERS Modulate | Adjusted Post-Task Score | Autistic Dyads | -0.07 | -0.07 | 0.000000 | 1.00 |
| S-DERS Modulate | Adjusted Post-Task Score | Mixed Dyads | 0.10 | 0.10 | 0.000000 | 1.00 |
| S-DERS Modulate | Dyad Contrast: Adjusted Score | Non-autistic vs Autistic | 0.05 | 0.05 | 0.000000 | 1.00 |
| S-DERS Modulate | Dyad Contrast: Adjusted Score | Non-autistic vs Mixed | -0.12 | -0.12 | 0.000000 | 1.00 |
| S-DERS Modulate | Dyad Contrast: Adjusted Score | Autistic vs Mixed | -0.17 | -0.17 | 0.000000 | 1.00 |
| S-DERS Modulate | Synchrony Slope | Non-Autistic Dyads | 0.05 | 0.05 | 0.000000 | 1.00 |
| S-DERS Modulate | Synchrony Slope | Autistic Dyads | 0.17 | 0.17 | 0.000000 | 1.00 |
| S-DERS Modulate | Synchrony Slope | Mixed Dyads | -0.40 | -0.40 | 0.000000 | 1.00 |
| S-DERS Modulate | Dyad Contrast: Slope | Non-autistic vs Autistic | -0.12 | -0.12 | 0.000000 | 1.00 |
| S-DERS Modulate | Dyad Contrast: Slope | Non-autistic vs Mixed | 0.45 | 0.45 | 0.000000 | 1.00 |
| S-DERS Modulate | Dyad Contrast: Slope | Autistic vs Mixed | 0.58 | 0.58 | 0.000000 | 1.00 |

*Note.* Comparison estimates are from models fit without the dyad random intercept when the corresponding primary model had a boundary fit. No comparison changed the conventional p < .05 decision status.

**Table 19**

*Practical-Equivalence Tests for Model-Based Estimands*

| Outcome | Estimand | Term | Estimate | SE | df | 90% CI | TOST p |
| --- | --- | --- | --- | --- | --- | --- | --- |
| Negative Affect | Adjusted Post-Task Score | Autistic Dyads | 0.14 | 0.17 | 25.38 | [-0.16, 0.43] | .583 |
| Negative Affect | Adjusted Post-Task Score | Mixed Dyads | -0.004 | 0.17 | 25.44 | [-0.29, 0.28] | .283 |
| Negative Affect | Adjusted Post-Task Score | Non-Autistic Dyads | -0.15 | 0.19 | 25.79 | [-0.47, 0.17] | .595 |
| Negative Affect | Synchrony Slope | Autistic Dyads | 0.01 | 0.17 | 26.78 | [-0.27, 0.30] | .302 |
| Negative Affect | Synchrony Slope | Mixed Dyads | -0.22 | 0.23 | 25.52 | [-0.62, 0.17] | .699 |
| Negative Affect | Synchrony Slope | Non-Autistic Dyads | -0.06 | 0.17 | 25.38 | [-0.35, 0.23] | .403 |
| Negative Affect | Dyad Contrast: Adjusted Score | Autistic vs Mixed | 0.14 | 0.24 | 25.42 | [-0.27, 0.55] | .566 |
| Negative Affect | Dyad Contrast: Adjusted Score | Non-autistic vs Autistic | -0.28 | 0.26 | 25.58 | [-0.72, 0.15] | .759 |
| Negative Affect | Dyad Contrast: Adjusted Score | Non-autistic vs Mixed | -0.14 | 0.25 | 25.81 | [-0.57, 0.29] | .566 |
| Negative Affect | Dyad Contrast: Slope | Autistic vs Mixed | 0.23 | 0.29 | 26.36 | [-0.26, 0.73] | .678 |
| Negative Affect | Dyad Contrast: Slope | Non-autistic vs Autistic | -0.07 | 0.24 | 26.10 | [-0.48, 0.33] | .451 |
| Negative Affect | Dyad Contrast: Slope | Non-autistic vs Mixed | 0.16 | 0.29 | 25.46 | [-0.32, 0.65] | .588 |
| Positive Affect | Adjusted Post-Task Score | Autistic Dyads | -0.21 | 0.16 | 26.35 | [-0.48, 0.06] | .762 |
| Positive Affect | Adjusted Post-Task Score | Mixed Dyads | -0.16 | 0.15 | 25.75 | [-0.41, 0.10] | .645 |
| Positive Affect | Adjusted Post-Task Score | Non-Autistic Dyads | 0.16 | 0.17 | 25.48 | [-0.13, 0.44] | .632 |
| Positive Affect | Synchrony Slope | Autistic Dyads | 0.04 | 0.15 | 25.72 | [-0.22, 0.29] | .334 |
| Positive Affect | Synchrony Slope | Mixed Dyads | 0.39 | 0.21 | 27.24 | [0.02, 0.75] | .904 |
| Positive Affect | Synchrony Slope | Non-Autistic Dyads | -0.21 | 0.15 | 25.51 | [-0.47, 0.04] | .771 |
| Positive Affect | Dyad Contrast: Adjusted Score | Autistic vs Mixed | -0.06 | 0.22 | 26.55 | [-0.43, 0.32] | .425 |
| Positive Affect | Dyad Contrast: Adjusted Score | Non-autistic vs Autistic | 0.37 | 0.23 | 25.81 | [-0.02, 0.76] | .875 |
| Positive Affect | Dyad Contrast: Adjusted Score | Non-autistic vs Mixed | 0.31 | 0.23 | 25.64 | [-0.07, 0.70] | .824 |
| Positive Affect | Dyad Contrast: Slope | Autistic vs Mixed | -0.35 | 0.26 | 26.12 | [-0.79, 0.09] | .829 |
| Positive Affect | Dyad Contrast: Slope | Non-autistic vs Autistic | -0.25 | 0.21 | 25.70 | [-0.61, 0.11] | .758 |
| Positive Affect | Dyad Contrast: Slope | Non-autistic vs Mixed | -0.60 | 0.26 | 26.88 | [-1.05, -0.15] | .966 |
| S-DERS Modulate | Adjusted Post-Task Score | Autistic Dyads | -0.07 | 0.16 | 25.45 | [-0.34, 0.20] | .423 |
| S-DERS Modulate | Adjusted Post-Task Score | Mixed Dyads | 0.10 | 0.15 | 25.46 | [-0.16, 0.36] | .491 |
| S-DERS Modulate | Adjusted Post-Task Score | Non-Autistic Dyads | -0.02 | 0.17 | 26.22 | [-0.32, 0.28] | .324 |
| S-DERS Modulate | Synchrony Slope | Autistic Dyads | 0.17 | 0.15 | 25.29 | [-0.08, 0.43] | .685 |
| S-DERS Modulate | Synchrony Slope | Mixed Dyads | -0.40 | 0.21 | 25.38 | [-0.77, -0.04] | .919 |
| S-DERS Modulate | Synchrony Slope | Non-Autistic Dyads | 0.05 | 0.15 | 25.34 | [-0.21, 0.31] | .369 |
| S-DERS Modulate | Dyad Contrast: Adjusted Score | Autistic vs Mixed | -0.17 | 0.22 | 25.25 | [-0.54, 0.21] | .616 |
| S-DERS Modulate | Dyad Contrast: Adjusted Score | Non-autistic vs Autistic | 0.05 | 0.24 | 26.30 | [-0.36, 0.45] | .416 |
| S-DERS Modulate | Dyad Contrast: Adjusted Score | Non-autistic vs Mixed | -0.12 | 0.23 | 26.33 | [-0.51, 0.28] | .527 |
| S-DERS Modulate | Dyad Contrast: Slope | Autistic vs Mixed | 0.58 | 0.26 | 25.43 | [0.13, 1.02] | .961 |
| S-DERS Modulate | Dyad Contrast: Slope | Non-autistic vs Autistic | -0.12 | 0.21 | 25.38 | [-0.49, 0.24] | .546 |
| S-DERS Modulate | Dyad Contrast: Slope | Non-autistic vs Mixed | 0.45 | 0.26 | 25.26 | [0.007, 0.90] | .906 |
| S-DERS Total | Adjusted Post-Task Score | Autistic Dyads | 0.05 | 0.15 | 26.05 | [-0.20, 0.30] | .372 |
| S-DERS Total | Adjusted Post-Task Score | Mixed Dyads | 0.02 | 0.14 | 25.36 | [-0.22, 0.25] | .277 |
| S-DERS Total | Adjusted Post-Task Score | Non-Autistic Dyads | -0.03 | 0.16 | 26.82 | [-0.30, 0.25] | .329 |
| S-DERS Total | Synchrony Slope | Autistic Dyads | 0.10 | 0.14 | 25.45 | [-0.14, 0.33] | .489 |
| S-DERS Total | Synchrony Slope | Mixed Dyads | -0.35 | 0.20 | 26.43 | [-0.69, -0.01] | .891 |
| S-DERS Total | Synchrony Slope | Non-Autistic Dyads | 0.06 | 0.14 | 25.44 | [-0.18, 0.30] | .399 |
| S-DERS Total | Dyad Contrast: Adjusted Score | Autistic vs Mixed | 0.03 | 0.20 | 25.49 | [-0.31, 0.38] | .374 |
| S-DERS Total | Dyad Contrast: Adjusted Score | Non-autistic vs Autistic | -0.08 | 0.22 | 27.51 | [-0.46, 0.30] | .463 |
| S-DERS Total | Dyad Contrast: Adjusted Score | Non-autistic vs Mixed | -0.04 | 0.21 | 26.51 | [-0.41, 0.32] | .399 |
| S-DERS Total | Dyad Contrast: Slope | Autistic vs Mixed | 0.45 | 0.24 | 26.50 | [0.03, 0.86] | .917 |
| S-DERS Total | Dyad Contrast: Slope | Non-autistic vs Autistic | -0.03 | 0.20 | 25.60 | [-0.37, 0.30] | .368 |
| S-DERS Total | Dyad Contrast: Slope | Non-autistic vs Mixed | 0.41 | 0.24 | 25.69 | [0.001, 0.82] | .897 |
| Interpersonal Synchrony | Std. Dyad Contrast | Autistic vs Mixed | 0.17 | 0.20 | 28.95 | [-0.17, 0.52] | .639 |
| Interpersonal Synchrony | Std. Dyad Contrast | Non-autistic vs Autistic | -0.56 | 0.20 | 29.06 | [-0.90, -0.22] | .986 |
| Interpersonal Synchrony | Std. Dyad Contrast | Non-autistic vs Mixed | -0.39 | 0.20 | 28.95 | [-0.74, -0.04] | .916 |
| Interpersonal Synchrony | Std. Mean | Autistic Dyads | 0.25 | 0.14 | 29.06 | [0.007, 0.49] | .848 |
| Interpersonal Synchrony | Std. Mean | Mixed Dyads | 0.07 | 0.15 | 28.85 | [-0.18, 0.33] | .431 |
| Interpersonal Synchrony | Std. Mean | Non-Autistic Dyads | -0.32 | 0.14 | 29.06 | [-0.56, -0.08] | .931 |

*Note.* TOST = two one-sided tests; CI = confidence interval; SE = standard error. Equivalence bounds were -0.10 and 0.10 standardized units for all estimands. The 90% CI corresponds to the alpha = .05 TOST decision rule. No tested estimand met the practical-equivalence criterion under these bounds.

**Table 20**

*80 Percent Minimum Detectable Effects for Model-Based Estimands*

| Outcome | Estimand | Term | SE | df | MDE |
| --- | --- | --- | --- | --- | --- |
| Negative Affect | Synchrony Slope | Autistic Dyads | 0.17 | 26.78 | 0.49 |
| Negative Affect | Synchrony Slope | Mixed Dyads | 0.23 | 25.52 | 0.67 |
| Negative Affect | Synchrony Slope | Non-Autistic Dyads | 0.17 | 25.38 | 0.49 |
| Negative Affect | Dyad Contrast: Adjusted Score | Autistic vs Mixed | 0.24 | 25.42 | 0.70 |
| Negative Affect | Dyad Contrast: Adjusted Score | Non-autistic vs Autistic | 0.26 | 25.58 | 0.74 |
| Negative Affect | Dyad Contrast: Adjusted Score | Non-autistic vs Mixed | 0.25 | 25.81 | 0.73 |
| Negative Affect | Dyad Contrast: Slope | Autistic vs Mixed | 0.29 | 26.36 | 0.84 |
| Negative Affect | Dyad Contrast: Slope | Non-autistic vs Autistic | 0.24 | 26.10 | 0.69 |
| Negative Affect | Dyad Contrast: Slope | Non-autistic vs Mixed | 0.29 | 25.46 | 0.83 |
| Positive Affect | Synchrony Slope | Autistic Dyads | 0.15 | 25.72 | 0.43 |
| Positive Affect | Synchrony Slope | Mixed Dyads | 0.21 | 27.24 | 0.62 |
| Positive Affect | Synchrony Slope | Non-Autistic Dyads | 0.15 | 25.51 | 0.44 |
| Positive Affect | Dyad Contrast: Adjusted Score | Autistic vs Mixed | 0.22 | 26.55 | 0.64 |
| Positive Affect | Dyad Contrast: Adjusted Score | Non-autistic vs Autistic | 0.23 | 25.81 | 0.67 |
| Positive Affect | Dyad Contrast: Adjusted Score | Non-autistic vs Mixed | 0.23 | 25.64 | 0.66 |
| Positive Affect | Dyad Contrast: Slope | Autistic vs Mixed | 0.26 | 26.12 | 0.75 |
| Positive Affect | Dyad Contrast: Slope | Non-autistic vs Autistic | 0.21 | 25.70 | 0.62 |
| Positive Affect | Dyad Contrast: Slope | Non-autistic vs Mixed | 0.26 | 26.88 | 0.76 |
| S-DERS Modulate | Synchrony Slope | Autistic Dyads | 0.15 | 25.29 | 0.44 |
| S-DERS Modulate | Synchrony Slope | Mixed Dyads | 0.21 | 25.38 | 0.62 |
| S-DERS Modulate | Synchrony Slope | Non-Autistic Dyads | 0.15 | 25.34 | 0.45 |
| S-DERS Modulate | Dyad Contrast: Adjusted Score | Autistic vs Mixed | 0.22 | 25.25 | 0.64 |
| S-DERS Modulate | Dyad Contrast: Adjusted Score | Non-autistic vs Autistic | 0.24 | 26.30 | 0.69 |
| S-DERS Modulate | Dyad Contrast: Adjusted Score | Non-autistic vs Mixed | 0.23 | 26.33 | 0.68 |
| S-DERS Modulate | Dyad Contrast: Slope | Autistic vs Mixed | 0.26 | 25.43 | 0.76 |
| S-DERS Modulate | Dyad Contrast: Slope | Non-autistic vs Autistic | 0.21 | 25.38 | 0.63 |
| S-DERS Modulate | Dyad Contrast: Slope | Non-autistic vs Mixed | 0.26 | 25.26 | 0.76 |
| S-DERS Total | Synchrony Slope | Autistic Dyads | 0.14 | 25.45 | 0.40 |
| S-DERS Total | Synchrony Slope | Mixed Dyads | 0.20 | 26.43 | 0.58 |
| S-DERS Total | Synchrony Slope | Non-Autistic Dyads | 0.14 | 25.44 | 0.41 |
| S-DERS Total | Dyad Contrast: Adjusted Score | Autistic vs Mixed | 0.20 | 25.49 | 0.59 |
| S-DERS Total | Dyad Contrast: Adjusted Score | Non-autistic vs Autistic | 0.22 | 27.51 | 0.65 |
| S-DERS Total | Dyad Contrast: Adjusted Score | Non-autistic vs Mixed | 0.21 | 26.51 | 0.62 |
| S-DERS Total | Dyad Contrast: Slope | Autistic vs Mixed | 0.24 | 26.50 | 0.71 |
| S-DERS Total | Dyad Contrast: Slope | Non-autistic vs Autistic | 0.20 | 25.60 | 0.58 |
| S-DERS Total | Dyad Contrast: Slope | Non-autistic vs Mixed | 0.24 | 25.69 | 0.70 |
| Interpersonal Synchrony | Std. Dyad Contrast | Autistic vs Mixed | 0.20 | 28.95 | 0.59 |
| Interpersonal Synchrony | Std. Dyad Contrast | Non-autistic vs Autistic | 0.20 | 29.06 | 0.58 |
| Interpersonal Synchrony | Std. Dyad Contrast | Non-autistic vs Mixed | 0.20 | 28.95 | 0.59 |

*Note.* Minimum detectable effects were computed from model standard errors and degrees of freedom for two-sided alpha .05 tests with 80% target power. MDEs for synchrony-slope rows are in standardized post-task score units per 1 SD interpersonal synchrony. MDEs for adjusted-score dyad contrasts are in standardized post-task score units. MDEs for standardized dyad contrasts are in standardized interpersonal synchrony units.

## 1.5. Alexithymia-Adjusted Affect Models

**Table 21**

*Estimated Coefficients for the Alexithymia-Adjusted PANAS Models*

|  | | | *95% CI* | |  |
| --- | --- | --- | --- | --- | --- |
| Effect | Estimate | *SE* | *LL* | *UL* | *p* |
| ***Positive Affect*** |  |  |  |  |  |
| **Fixed effects** |  |  |  |  |  |
| Intercept | .10 | .18 | -.25 | .46 | .568 |
| Pre-Task Score (z) | .66*** | .09 | .47 | .84 | < .001 |
| Dyad Type: ASD-ASD | -.27 | .25 | -.78 | .23 | .281 |
| Dyad Type: ASD-NT | -.24 | .24 | -.71 | .24 | .328 |
| Alexithymia Total (z) | -.10 | .10 | -.29 | .10 | .339 |
| Interpersonal Synchrony Slope: Non-autistic | -.22 | .15 | -.52 | .09 | .157 |
| Interpersonal Synchrony Slope: Autistic | .03 | .15 | -.27 | .33 | .848 |
| Interpersonal Synchrony Slope: Mixed | .35 | .22 | -.09 | .78 | .116 |
| **Random effects** |  |  |  |  |  |
| Random intercept SD (Dyad Number) | .00 |  |  |  |  |
| Residual SD | .65 |  |  |  |  |
| **Model fit** |  |  |  |  |  |
| Observations | 64 |  |  |  |  |
| AIC | 156.68 |  |  |  |  |
| BIC | 178.27 |  |  |  |  |
| ***Negative Affect*** |  |  |  |  |  |
| **Fixed effects** |  |  |  |  |  |
| Intercept | -.12 | .19 | -.52 | .27 | .529 |
| Pre-Task Score (z) | .73*** | .09 | .55 | .91 | < .001 |
| Dyad Type: Autistic | .24 | .27 | -.32 | .80 | .394 |
| Dyad Type: Mixed | .11 | .26 | -.42 | .65 | .666 |
| Alexithymia Total (z) | .04 | .09 | -.15 | .23 | .642 |
| Interpersonal Synchrony Slope: Non-autistic | -.06 | .17 | -.41 | .29 | .738 |
| Interpersonal Synchrony Slope: Autistic | .02 | .17 | -.33 | .37 | .891 |
| Interpersonal Synchrony Slope: Mixed | -.20 | .24 | -.69 | .29 | .407 |
| **Random effects** |  |  |  |  |  |
| Random intercept SD (Dyad Number) | .32 |  |  |  |  |
| Residual SD | .58 |  |  |  |  |
| **Model fit** |  |  |  |  |  |
| Observations | 64 |  |  |  |  |
| AIC | 154.97 |  |  |  |  |
| BIC | 176.56 |  |  |  |  |

*Note.* CI = confidence interval; LL = lower limit; UL = upper limit; SD = standard deviation. Fixed-effect estimates are reported relative to the NT-NT dyad group, with post-task scores adjusted for the corresponding pre-task score and alexithymia. Random-effect rows report estimated standard deviations. Model fit rows report the number of observations, Akaike information criterion (AIC), and Bayesian information criterion (BIC).

* p < .05. ** p < .01. *** p < .001.

**Table 22**

*Alexithymia-Adjusted PANAS Post-Task Scores by Dyad Type*

| outcome | dyad | estimate | SE | 95% CI | t | p |
| --- | --- | --- | --- | --- | --- | --- |
| Positive Affect | Non-autistic | 0.10 | 0.18 | [-0.26, 0.47] | 0.57 | .572 |
| Positive Affect | Autistic | -0.17 | 0.16 | [-0.51, 0.17] | -1.05 | .305 |
| Positive Affect | Mixed | -0.13 | 0.15 | [-0.45, 0.18] | -0.88 | .385 |
| Negative Affect | Non-autistic | -0.12 | 0.19 | [-0.52, 0.28] | -0.64 | .529 |
| Negative Affect | Autistic | 0.11 | 0.18 | [-0.26, 0.49] | 0.62 | .539 |
| Negative Affect | Mixed | -0.01 | 0.17 | [-0.36, 0.34] | -0.06 | .951 |

*Note.* Estimates are baseline- and alexithymia-adjusted post-task PANAS scores at average interpersonal synchrony, average pre-task PANAS score, and average alexithymia.

**Table 23**

*Contrasts of Alexithymia-Adjusted PANAS Post-Task Scores by Dyad Type*

| outcome | contrast | estimate | SE | 95% CI | t | p |
| --- | --- | --- | --- | --- | --- | --- |
| Positive Affect | Non-autistic vs Autistic | 0.27 | 0.25 | [-0.35, 0.90] | 1.08 | .531 |
| Positive Affect | Non-autistic vs Mixed | 0.24 | 0.24 | [-0.36, 0.83] | 0.98 | .593 |
| Positive Affect | Autistic vs Mixed | -0.04 | 0.22 | [-0.59, 0.51] | -0.17 | .984 |
| Negative Affect | Non-autistic vs Autistic | -0.24 | 0.28 | [-0.92, 0.44] | -0.86 | .668 |
| Negative Affect | Non-autistic vs Mixed | -0.11 | 0.26 | [-0.76, 0.53] | -0.44 | .901 |
| Negative Affect | Autistic vs Mixed | 0.12 | 0.25 | [-0.49, 0.73] | 0.51 | .869 |

*Note.* Estimates are pairwise dyad-type contrasts in baseline- and alexithymia-adjusted post-task PANAS scores.

**Table 24**

*Interpersonal Synchrony Slopes for Alexithymia-Adjusted PANAS Post-Task Scores*

| outcome | dyad | estimate | SE | 95% CI | t | p |
| --- | --- | --- | --- | --- | --- | --- |
| Positive Affect | Non-autistic | -0.22 | 0.15 | [-0.53, 0.10] | -1.43 | .164 |
| Positive Affect | Autistic | 0.03 | 0.15 | [-0.28, 0.33] | 0.19 | .849 |
| Positive Affect | Mixed | 0.35 | 0.22 | [-0.10, 0.79] | 1.59 | .122 |
| Negative Affect | Non-autistic | -0.06 | 0.17 | [-0.41, 0.29] | -0.34 | .738 |
| Negative Affect | Autistic | 0.02 | 0.17 | [-0.33, 0.37] | 0.14 | .892 |
| Negative Affect | Mixed | -0.20 | 0.24 | [-0.69, 0.29] | -0.84 | .408 |

*Note.* Estimates are simple slopes relating interpersonal synchrony to baseline- and alexithymia-adjusted post-task PANAS scores within each dyad type.

**Table 25**

*Contrasts of Interpersonal Synchrony Slopes for Alexithymia-Adjusted PANAS Post-Task Scores*

| outcome | contrast | estimate | SE | 95% CI | t | p |
| --- | --- | --- | --- | --- | --- | --- |
| Positive Affect | Non-autistic vs Autistic | -0.25 | 0.21 | [-0.77, 0.28] | -1.16 | .487 |
| Positive Affect | Non-autistic vs Mixed | -0.56 | 0.27 | [-1.22, 0.09] | -2.12 | .103 |
| Positive Affect | Autistic vs Mixed | -0.32 | 0.26 | [-0.96, 0.33] | -1.23 | .449 |
| Negative Affect | Non-autistic vs Autistic | -0.08 | 0.24 | [-0.68, 0.52] | -0.34 | .940 |
| Negative Affect | Non-autistic vs Mixed | 0.14 | 0.29 | [-0.58, 0.87] | 0.49 | .877 |
| Negative Affect | Autistic vs Mixed | 0.22 | 0.29 | [-0.50, 0.95] | 0.77 | .725 |

*Note.* Estimates are pairwise dyad-type contrasts in the simple slopes relating interpersonal synchrony to baseline- and alexithymia-adjusted post-task PANAS scores.

**Table 26**

*Covariate Descriptives*

| Variable | Grouping | Group | n | Mean | SD | Min | Max |
| --- | --- | --- | --- | --- | --- | --- | --- |
| Alexithymia Total | Participant Type | Non-ASC | 32 | 38.12 | 8.42 | 27.00 | 56.00 |
| Alexithymia Total | Participant Type | ASC | 32 | 51.19 | 8.75 | 32.00 | 68.00 |
| Sport Level | Participant Type | Non-ASC | 30 | 0.70 | 0.92 | 0.00 | 2.00 |
| Sport Level | Participant Type | ASC | 30 | 0.50 | 0.86 | 0.00 | 2.00 |
| Mean Sport Level | Dyad Type | Non-Autistic Dyads | 10 | 0.70 | 0.54 | 0.00 | 1.50 |
| Mean Sport Level | Dyad Type | Autistic Dyads | 10 | 0.60 | 0.70 | 0.00 | 2.00 |
| Mean Sport Level | Dyad Type | Mixed Dyads | 9 | 0.50 | 0.71 | 0.00 | 2.00 |

*Note.* Descriptive statistics summarize alexithymia and self-reported sport activity by analysis group.

**Table 27**

*Sport Activity Group Comparisons*

| Comparison | Grouping | n | Statistic | df | p |
| --- | --- | --- | --- | --- | --- |
| Wilcoxon Rank Sum | Participant Type | 60 | 504.00 |  | .339 |
| Kruskal Wallis | Dyad Type | 29 | 0.91 | 2.00 | .635 |

*Note.* Participant-type and dyad-type sport activity comparisons are reported as descriptive covariate context.

Figure S1
Observed Pre- and Posttask S-DERS Scores by Dyad Type

*Note* . Points represent individual participants' observed State Difficulties in Emotion Regulation Scale (S-DERS) scores before and after the body coordination task; gray lines connect repeated observations from the same participant. Boxplots show medians and interquartile ranges, with whiskers extending to 1.5 interquartile ranges. Columns show dyad type: non-autistic dyads, autistic dyads, and mixed dyads. Point shape indicates participant type. Higher S-DERS scores indicate greater difficulties in emotion regulation. S-DERS Modulate refers to the modulate subscale.

Figure S2

Observed Pre- and Posttask PANAS Scores by Dyad Type

 *Note*. Points represent individual participants' observed Positive and Negative Affect Schedule (PANAS) scores before and after the body coordination task; gray lines connect repeated observations from the same participant. Boxplots show medians and interquartile ranges, with whiskers extending to 1.5 interquartile ranges. Columns show dyad type: non-autistic dyads, autistic dyads, and mixed dyads. Point shape indicates participant type. Higher PANAS scores indicate greater positive or negative affect, respectively.

**German version of S-DERS**

S-DERS	CODEWORT ________________

Anleitung: Bitte lesen Sie jede Aussage und geben Sie an, wie sehr sie auf IHRE EMOTIONEN JETZT zutrifft.

| 1 | 2 | 3 | 4 | 5 |
| --- | --- | --- | --- | --- |
| Überhaupt nicht | Ein wenig | Mittelmäßig | Sehr stark | Vollkommen |

______	1) Ich fühle mich schuldig, dass ich mich so fühle.

______	2) Ich achte darauf, wie ich mich fühle.

______	3) Ich fühle mich außer Kontrolle.

______	4) Es ist mir peinlich, so zu fühlen.

______	5) Ich fühle mich sehr schlecht mit mir selbst.

______	6) Ich erkenne meine Gefühle an.

______	7) Ich habe keine Ahnung, wie ich mich fühle.

______	8) Ich schäme mich für mich selbst, dass ich mich so fühle.

______	9) Ich habe Schwierigkeiten, die Dinge zu tun, die ich im Moment tun muss.

______	10) Ich glaube, dass ich mich noch lange so fühlen werde.

______	11) Mich interessiert, was ich fühle.

______	12) Ich bin wütend auf mich selbst, weil ich so fühle.

______	13) Ich habe Schwierigkeiten, mein Verhalten zu kontrollieren.

______	14) Ich bin verwirrt, wie ich mich fühle.

______	15) Ich glaube, dass ich am Ende sehr deprimiert sein werde.

______	16) Ich nehme mir Zeit, um herauszufinden, was ich wirklich fühle.

______	17) Meine Emotionen sind außer Kontrolle geraten.

______	18) Ich bin irritiert von mir selbst, dass ich mich so fühle.

______	19) Ich glaube, dass meine Gefühle berechtigt und wichtig sind.

______	20) Ich fühle mich wie ein schwacher Mensch, weil ich so fühle.

______	21) Meine Gefühle fühlen sich überwältigend an.
